# Targeted DNA degradation using a CRISPR device stably carried in the host genome

**DOI:** 10.1038/ncomms7989

**Published:** 2015-05-19

**Authors:** Brian J. Caliando, Christopher A. Voigt

**Affiliations:** 1 Synthetic Biology Center, Department of Biological Engineering, Massachusetts Institute of Technology, 77 Massachusetts Avenue, Cambridge, Massachusetts 02139, USA

## Abstract

Once an engineered organism completes its task, it is useful to degrade the associated DNA to reduce environmental release and protect intellectual property. Here we present a genetically encoded device (DNAi) that responds to a transcriptional input and degrades user-defined DNA. This enables engineered regions to be obscured when the cell enters a new environment. DNAi is based on type-IE CRISPR biochemistry and a synthetic CRISPR array defines the DNA target(s). When the input is on, plasmid DNA is degraded 10^8^-fold. When the genome is targeted, this causes cell death, reducing viable cells by a factor of 10^8^. Further, the CRISPR nuclease can direct degradation to specific genomic regions (for example, engineered or inserted DNA), which could be used to complicate recovery and sequencing efforts. DNAi can be stably carried in an engineered organism, with no impact on cell growth, plasmid stability or DNAi inducibility even after passaging for >2 months.

## Abstract (web summary)

The ability to contain and destroy synthetically engineered microorganisms is an important consideration with environmental, industrial and intellectual property implications. Here Caliando *et al*. design and demonstrate a stably integrated CRISPR-based system for targeted DNA destruction.

## Graphical abstract

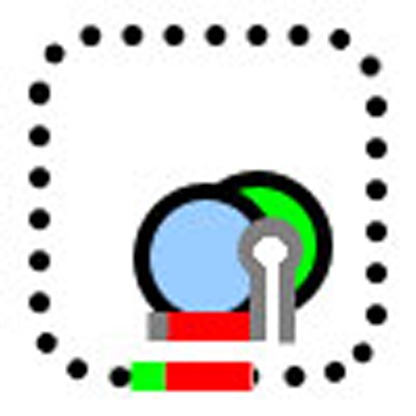

The ability to programme cells to eliminate engineered DNA at a defined time point or change in environments would benefit many applications in biotechnology. For example, after bio-manufacturing a chemical, cells could be programmed to degrade their DNA at the end of the process or when they are removed from a defined medium. This would aid the protection of sequence information as a trade secret, make it easier to remove DNA contamination from a product, reduce the cost of biomass disposal and decrease the amount of DNA in the environment after an accidental release. There are similar needs for ‘out of the bioreactor' applications, such as using engineered cells as living therapeutics (for example, in the gut microbiome) or in forming associations with crop plants in a field^1^. In these cases, it is impossible to recollect cells for disposal, so they need to be programmed to degrade their own DNA when they leave a defined environment or after a defined time period.

Various genetic switches have been developed that induce cell death^2^. The actuation is based on toxic proteins that are under tight regulatory control to avoid background expression^3^,^4^ and have been combined with synthetic genetic circuits that trigger cell death under a shift in environmental conditions or change in cell state^5^,^6^,^7^,^8^,^9^. Inducing cell death does not address the problem of the release of DNA, which persists after cells die or are killed. Indeed, waste streams from fermenters are rich with recombinant DNA, even when the cells have been inactivated by heat, pH and antibiotics^10^ and, in fact, these methods of rapid cell death exacerbate the release of extracellular DNA^11^. The waste biomass of engineered microbes is often used as agricultural fertilizer (for example, NovoGro), and this has been shown to contain significant amounts of DNA^10^. Furthermore, DNA molecules are stable and, once introduced, plasmid and genomic DNA can be recovered from environmental samples via PCR for 1–5 months^11^,^12^ and is likely to be detectable longer with advances in deep sequencing^13^. In one study, 35% of plasmid DNA molecules that were exposed to the extreme heat and pressures of atmospheric re-entry on the surface of a rocket still retained their biological function^14^.

The ease of recovering DNA from environmental samples poses a challenge for the biotech industry. First, the value and competitive advantage of a company is largely in its engineered DNA constructs and strains and these involve large financial investments and development time. Protecting this information as a trade secret is nearly impossible when samples can be easily recovered from a waste stream (or as a contaminant in a consumer product), sequenced and then rebuilt using chemical DNA synthesis. Therefore, the copying of the products of genetic engineering no longer requires the transfer or theft of intact DNA molecules or living cells. In the future, it may be possible to access complete organisms through the synthesis and transplantation of entire genomes^15^,^16^,^17^.

Once DNA is stably introduced into cells, removing it is tedious and difficult to scale to an industrial process. The traditional way to remove plasmids is to ‘cure' them by culturing on non-selecting media over multiple generations^18^. Another method is to express nonspecific DNA nucleases, which efficiently cause cell death (1:10^5^, ref. 8), but their nonspecificity can severely inhibit cell growth even when maintained in the uninduced state^2^. DNA can also be removed via downstream processing of the fermentation product or waste streams. This is necessary for the production of therapeutic proteins and DNA (for gene therapy and vaccines), where the US Food and Drug Administration enforces allowable limits for contaminant DNA (10 ng per dose)^19^,^20^. This either requires high-performance purification methods and/or removal of DNA specifically via ion exchange columns or membrane filtration, all of which are expensive and difficult to implement^19^,^20^,^21^.

The RNA guide-directed DNAse machinery from type-I, -II and -IIIA CRISPR defense systems^22^ provides a means to remove DNA by directing nuclease activity to target sequences. CRISPR is an ‘immune system' for prokaryotes that prevents transduction by phage and conjugative plasmids by targeting their DNA for degradation^22^,^23^,^24^. The targeted DNA sequences are specified by the CRISPR array, which is a series of ∼30–40 bp spacers separated by short palindromic repeats^22^,^23^,^24^. The array is transcribed as a pre-crRNA and is processed into shorter crRNAs that associate with the Cas protein complex to target complementary DNA sequences known as proto-spacers^25^,^26^,^27^,^28^,^29^,^30^,^31^,^32^,^33^. These proto-spacer targets must also have an additional neighbouring sequence known as a proto-spacer adjacent motif (PAM) that is required for target recognition^33^,^34^,^35^,^36^,^37^,^38^. After binding, a Cas protein complex serves as a DNA endonuclease to cut both strands at the target^32^,^33^ and subsequent DNA degradation occurs via exonuclease activity^32^. The RNA-guiding capability of the Cas protein(s) has been widely applied to problems in biotechnology, including genome editing and synthetic regulation^39^,^40^,^41^,^42^,^43^,^44^,^45^.

There are several advantages to using the CRISPR nuclease to remove intracellular DNA. First, the guide sequences can be used to direct nuclease activity to user-defined DNA targets with high specificity. This can be used to knock out plasmids without harming the host or to target specific sensitive regions of the genome. Second, it can target multiple sequences in parallel. Finally, it has shown to be functional in many cell types, including eukaryotes and higher organisms. We selected the Type-IE CRISPR system native to K-type *Escherichia coli*, which has been shown to inhibit phage infection^25^,^38^,^46^,^47^, cure plasmids 32,44,48, prevent conjugal element transfer^49^ and kill cells^45^,^50^.

Here, we describe the construction of a genetic device (DNAi) whose input is a transcriptional signal and whose output is the CRISPR-mediated targeted degradation of DNA (Fig. 1a). Defining a transcriptional input enables it to be connected to the output of inducible systems, environmental sensors or genetic circuits^51^. Modularized component design enables rapid reprogramming of the device's output to degrade any user-specified set of DNA targets. The device stably maintains all of the necessary components for DNAi-mediated nuclease activity and, on induction, degrades the target DNA while leaving non-targeted DNA unaffected. This requires the device to retain a large dynamic range when induced, yet have almost no basal activity when carried in the host genome in the uninduced state. We demonstrate that the DNAi device is effective at knocking out plasmid targets with a wide range of copy numbers quickly and efficiently. We also show that DNAi activity renders the targeted DNA sequence information more difficult to recover via PCR amplification, that alternative PAM sequences can be used to tune the device's kinetic profile, and that targeting DNAi activity to the host chromosome enables highly efficient inducible cell killing. Furthermore, when passaged in the off state, the device does not impose an appreciable growth burden on the host, remains stable and active over many generations and does not affect protein expression from the target DNA before induction.

## Results

### Design and optimization of the DNAi device

**Figure 1:**
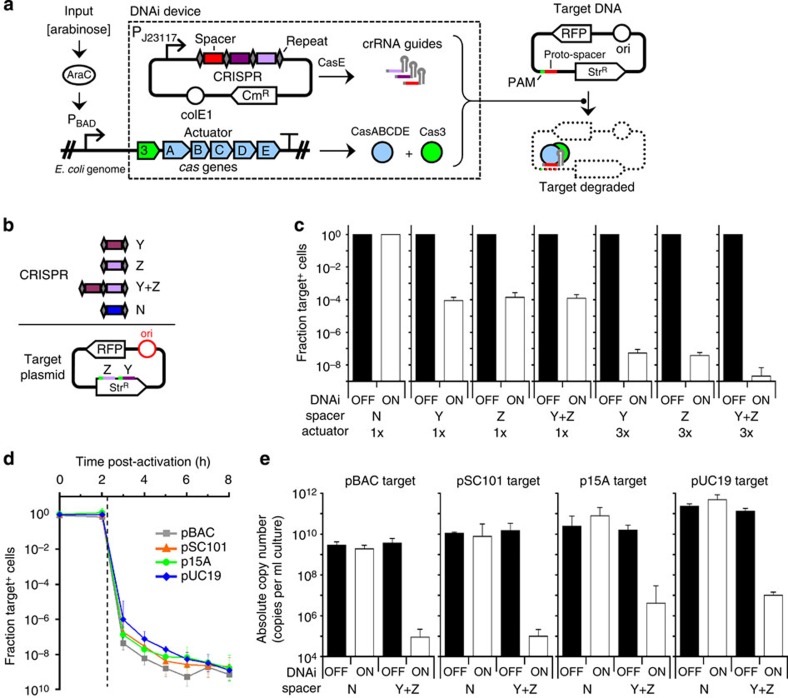
Characterization of the DNAi device. (**a**) A schematic of the device (dashed box) is shown. The CRISPR array is transcribed from a constitutive promoter (P_J23117_). (**b**) The Y and Z spacers are designed to be internal to the Str^R^ gene carried on the targeted plasmid. The N spacer is a control whose sequence is not present in the targeted plasmid or genome. The spacer sequences and the PAMs associated with their corresponding proto-spacers are presented in Supplementary Table 1. (**c**) Data for the knockout of target plasmids are shown for different spacers and numbers of actuators carried in the genome (1x and 3x). The fraction of cells that retain the pSC101 origin pTAR(S) target plasmid (Target^+^) is shown in the OFF (repressed by 0.5% glucose) and ON (2 mM arabinose) states. The OFF data and the 1x/N ON data were obtained using the cytometry assay and the remainder were obtained by the plate-based assay (Methods). (**d**) A time course is shown for the knockout of target plasmids containing different origins of replication. The inducer (2 mM arabinose) is added at *t*=0 h and aliquots of each sample are removed over time and analysed using the plating assay to determine the fraction retaining the target plasmid (Methods). The vertical dashed line indicates *t*=2.25 h, the time at which the P_BAD_ promoter activates to initiate the synthesis of DNAi actuator components (Supplementary Fig. 5a). All of the data were gathered using the 3x DNAi device and the Y+Z dual spacers. When the target contains the pUC19 origin, the plasmid containing the targeting array was changed to a compatible RSF origin (pCR-YZ* and pCR-N*). (**e**) The recovery of target plasmid DNA sequences via PCR after the 8-h induction of DNAi as performed in **d** is quantified via qPCR and is given in terms of absolute copy number per millilitre of culture (ACN_culture_; Methods). All data represent the average of three independent experiments performed on different days and the error bars are the s.d.

The design for the DNAi device is shown in Fig. 1a. The transcriptional input induces an operon containing the *cas3* and *casABCDE* genes^25^. These genes are normally transcribed separately, but we combined them so that a single promoter could serve as the device input. The combined activities of Cas3 and the CasABCDE complex are analogous to Cas9, which is the basis for CRISPRi and genome editing. We chose the former because we hypothesized they could be less toxic in gram-negative hosts and unlike Cas9, Cas3 possesses 3′-to-5′ helicase^52^ and ssDNA exonulease activities^53^, which could enhance DNA degradation after the initial cleavage event. The DNA targeting information is in the form of a synthetic CRISPR array, composed of an alternating sequence of 29-bp repeats, which contain the recognition site for CasE-mediated crRNA processing, separated by 32-bp unique spacer sequences (Supplementary Table 1). The array is constitutively expressed from the weak P_J23117_ promoter. The *cas* actuator and targeting array can be carried on plasmids or the genome and remain functional (Supplementary Figs 1b, 2a, 3 and 4). For the work in this manuscript, the actuator is carried in the genome (Supplementary Table 2) to reduce background activity and the array is carried on a high-copy pUC19 plasmid to reduce loss-of-function mutations and to simplify the testing of different targets.

The input promoter serves as the master switch. We chose to characterize the device using the arabinose-inducible P_BAD_ promoter because it has a tight OFF state while maintaining a large dynamic range. Transcriptional activity from this promoter is turned on upon the addition of arabinose to the media and turned off with the addition of glucose. In theory, any promoter could be connected to the device that spans the required range of promoter activity (Supplementary Fig. 5). This could be an environmental sensor that responds to a particular media or growth phase, or the output of a genetic circuit that processes a more complex combination of sensory inputs. The activator for P_BAD_ is the AraC protein and a loss-of-function mutation in the corresponding gene would cause the DNAi device to fail. To hedge against this, up to three redundant copies of *araC* are expressed from the genome (Methods).

A plasmid-based target pTAR(S) was designed to test and optimize the device (Fig. 1b). Initially, a low copy origin was selected (pSC101, 10–12 copies) to increase sensitivity to leakage in the OFF state. The plasmid also contains a dual-phenotype reporter system (streptomycin resistance and red fluorescent protein, RFP) to quantify the presence or absence of the target plasmid in a population using either plate assays or flow cytometry (Methods). To test the DNAi device, we designated a proto-spacer target sequence (Y) that was already present in the wild-type Str^R^ coding region of the plasmid and possessed a native 5′-ATG PAM (Fig. 1b). A second spacer was designed as a negative control (N) that lacks any identity to the target plasmid or host genome. An *E. coli* strain containing a single genomic copy of the actuator (1x) was transformed with either the Y- or N-targeting plasmid. The cells were then grown in the presence (ON) or absence (OFF) of arabinose without antibiotic selection for the target plasmid (−spectinomycin, Methods). When the Y spacer is included, the plasmids are lost in all but 0.009% of the population (Fig. 1c). Importantly, this only occurs when the device is ON and no plasmid is lost when it is maintained in the OFF state. As expected, transcribing the off-target N spacer does not lead to any plasmid loss when the device is either in the ON or OFF state.

We sought to engineer the system to improve the efficiency at which the targeting plasmid is degraded. First, we changed the proto-spacer target to a different sequence within the Str^R^ gene (Z) that possesses a native 5′-AAG PAM. This target alone or in combination with the Y spacer yielded the same reduction in the target plasmid (Fig. 1c). However, when combined with a strain that includes the actuator at three locations in the genome (3x), the inclusion of two targets (Y+Z) improved the degradation 10^4^-fold, reducing the fraction of the cells carrying the plasmid to 2.1 × 10^−9^, which is equivalent to <30 colony forming units (c.f.u.) per ml. The increase in the number of copies of the actuator in the genome did not impact the level of the background activity, where >98% of the cells retained the plasmid when the actuator is carried in the OFF state. This is in contrast to what is observed when the actuator is carried on a plasmid system, where even low copy number backbones resulted in significant plasmid loss (Supplementary Fig. 4).

It is interesting that the additional spacer only improves efficiency for the 3x copy of the actuator, but does not improve efficiency when the strain carries only one copy (1x). To determine the cause of this effect, we characterized escape mutants to determine where mutations had occurred to disrupt DNAi activity (Supplementary Fig. 6). We found that for the 1x strain, the dominant type of escape mutation disrupted the actuator and only a relatively small fraction of mutations disrupted the spacer or proto-spacer target. Thus, adding a second spacer is of limited help. However, for the 3x strain, no escape mutations involved mutation of the actuator and all occurred in the spacer. Because mutation of the spacer is now limiting, the addition of a second spacer further improves the efficiency. Thus, under these conditions, the device's knockout efficiency is ultimately dictated by the single component that is most likely to fail. Adding redundancy lowers the probability that individual components will fail, but doing so merely re-establishes a new efficiency floor based on the next most failure-prone candidate.

Next, the optimized DNAi device was assayed for its ability to knock out plasmids with varying copy numbers. These target plasmids were constructed by substituting the pSC101 origin of replication (10–12 copies per genome, Fig. 1b) with either a pBAC (one to two copies per genome), p15A (20–30 copies per genome) or pUC19 (>500 copies per genome) origin^54^,^55^,^56^. The target plasmids were transformed into *E. coli* containing the 3x DNAi device and a targeting array with both the Y and Z spacers. Cells were induced with 2 mM arabinose and plasmid loss was measured periodically over 8 h (Fig. 1d). The dynamics were surprisingly independent of copy number. Once the P_BAD_ promoter turns on after ∼2 h (Supplementary Fig. 5a), there is a sudden drop in plasmid abundance followed by a slower decay. At steady-state, there is only a 1.8-fold difference in the residual presence of plasmid from the high-copy pUC19 (1.2 × 10^−9^) to the single-copy pBAC (7.1 × 10^−10^). We next examined the ability of DNAi to render the target plasmid DNA sequence more difficult to recover via PCR-based methods. After 8 h of induction, total plasmid DNA was isolated from each sample using a Qiagen miniprep, and quantitative PCR (qPCR) was used to quantify the amount of recoverable target DNA (Methods). Figure 1e shows how the absolute copy number of the recovered DNA sequence changes when the device is turned ON for the targets containing different origins of replication. Upon activation, the device dramatically diminishes the amount of DNA that can be recovered. The amount of residual DNA that remains after induction reflects the copy number of the target. Upon DNAi activation, all of the targets exhibit a fairly constant fold reduction of ∼10^4^. As expected, there is no appreciable reduction in the amount of PCR-recoverable DNA when the off-target N spacer is used. The DNAi device has no impact on the plasmid copy number when the device is carried in the OFF state. This can be seen for each target by comparing the amount of recoverable DNA between the N and Y+Z spacers for each of the plasmid targets (Fig. 1e).

**Figure 2:**
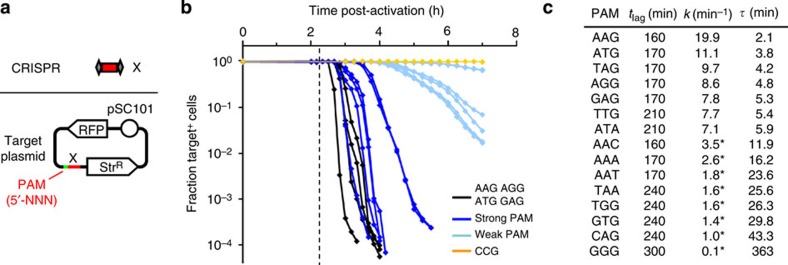
Effect of PAM sequence on DNAi device kinetics. (**a**) The X spacer is designed to be present in a non-coding region of the pPAM-*NNN*-RFP target plasmid series (pSC101 origin, Str^R^), where *NNN* corresponds to a different X-associated 3-bp PAM sequence of the form 5′-*NNN*-[Spacer X]. The X spacer sequence is presented in Supplementary Table 1. (**b**) The dynamics of plasmid loss are shown for each of the active PAM sequences. Data are for the 1x DNAi device, X spacer targeting plasmid, and a pSC101 target (RFP^+^, Str^R^) and reflect the fraction of cells in the ON state (2 mM arabinose) that retain the target plasmid (Target^+^) as determined by PAM kinetic assay (Methods). Black lines (AAG, AGG, ATG and GAG) correspond to the canonical PAM set identified by Westra *et al*.^32^ The vertical dashed line indicates *t*=2.25 h, the time at which the P_BAD_ promoter activates to initiate the synthesis of DNAi actuator components (Supplementary Fig. 5a). Details regarding the classification of strong versus weak PAMs are outlined in Supplementary Figs 7 and 8, and all canonical PAMs are classifiably strong PAMs. Target plasmid decay curves are truncated at the point in time where the fraction of Target^+^ cells reaches its minimum value. Error bars have been omitted for clarity but are shown in Supplementary Fig. 9. (**c**) Summary of kinetic parameters for plasmid loss experiments depicted in **b**. The length of the lag phase (*t*_lag_, in min) is defined as the time required for target retention to drop below 95%. The linear region(s) of log-scale decay curves from **b** were fit to the equation *y*=*A* × exp(−*kt*), where *t* is time (in min), *y* is the fraction retaining the plasmid and *A* is a constant. For curves that contain a kink, *k* was calculated for the first segment (identified by a ‘*'). The decay half-life (*τ*, in min) is calculated as 60*ln(2)/*k*. All data reflect the average of three independent experiments performed on different days.

In addition to spacer complementarity, DNAi also requires a valid 3-bp 5′ PAM sequence^35^,^37^,^38^. Others' experiments with phage transfection assays had identified four functional PAM sequences (5′-AAG, -AGG, -ATG and -GAG)^32^. We sought to determine whether additional 3-bp sequences could serve as active PAMs when used with our device. This would increase the range of potential target sequences, such that they would not be required to start with one of these four options. Serendipitously, we also discovered that the selection of the PAM sequence impacts the kinetics of DNA degradation. Preliminary experiments identified 10 strong PAMs (that include the known four) and five weak PAMs (Supplementary Figs 7 and 8), and so a set of RFP^+^ target plasmids (pSC101 origin, Str^R^) was constructed on the basis of the X proto-spacer coupled to each of these 15 PAM sequences, in addition to a 5′-CCG negative control (Fig. 2a). A plasmid knockout experiment was then performed with the 1x DNAi device, an X-targeting CRISPR plasmid and each of these PAM-coupled target plasmids (Fig. 2b, Supplementary Fig. 9, Methods). There was a wide spread in the kinetics of plasmid loss (Fig. 2c), with AAG corresponding to the fastest knockout and GGG corresponding to the slowest. The lag phase before initiation of target loss ranged from 160 min (AAG) to 300 min (GGG), and the associated half-lives of decay ranged over two orders of magnitude from 2.1 min (AAG) to 363 min (GGG). Collectively, this expanded PAM set will allow designers to target the greatest possible range of native sequences, will enable more comprehensive prediction of possible off-target interactions and will allow tailoring of DNAi kinetics from minutes to hours. This would enable different DNA sequences to be targeted at different times; for example, targeting plasmids first followed by the genome and the DNAi device itself.

### Genetic stability of the DNAi device

**Figure 3:**
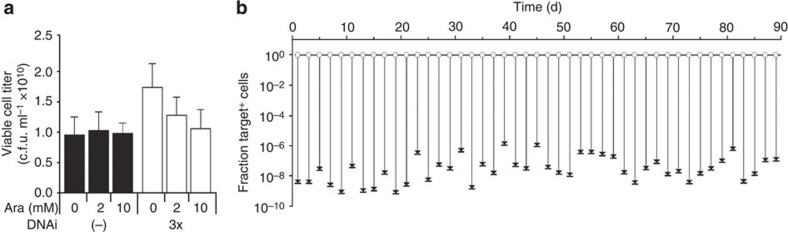
Growth impact and genetic stability of strains carrying the DNAi device. (**a**) The cell density is shown as a function of inducer (0, 2 and 10 mM Ara) for cells containing either the 3x DNAi device or no actuator, along with the Y+Z dual CRISPR targeting array (pCR-YZ, Cm^R^) and a target plasmid (pTAR(S), RFP^+^, Str^R^). Measurements of viable cell titres in c.f.u. per ml were made by plating serial dilutions of cultures onto solid media without selection for the target plasmid (Methods). The data represent the average of three independent experiments performed on different days and the error bars are the standard deviation. (**b**) The activity of the DNAi device was characterized periodically for 3 months. A strain containing the 3x DNAi device and the Y+Z dual spacer (pCR-YZ) and pSC101 target plasmid (pTAR(S)) was passaged every 12 h under conditions where the device is OFF (0.5% glucose; Methods). Every 2 days, aliquots were taken and analysed via the cytometry assay to determine the fraction of cells containing the target plasmid (Target^+^, white circles). These samples were then induced with 2 mM arabinose for 8 h and the fraction of the target plasmid was determined via plating assay (Methods; black dashes).

To be used effectively, the device must be silently carried in the OFF state and not slow growth, lower plasmid copy number or reduce the function of the engineered organism (for example, product yield). Toxicity could arise from the *cas* genes, where off-target nuclease activity could manifest as slower growth. To determine the toxicity of the ON state, we grew cells in different concentrations of inducer continuously for 8 h and then measured viable cell titre (in c.f.u. per ml) (Fig. 3a). The measurements were based on the 3x actuator and contained the Y+Z dual spacers and the targeting plasmid, which was being knocked out during the time course. Activity was well tolerated at the inducer concentrations used for the experiments in this manuscript (2 mM). Even upon maximal induction (10 mM), <40% reduction in culture density was observed. Furthermore, the rate of cell growth was minimally affected as well. When analogous growth rate measurements were performed comparing the 3x DNAi strain to the actuator-free strain, the maximal difference in doubling time was <1.5 min (Supplementary Fig. 10a). This is in contrast to when the *cas* genes are carried on a plasmid, where even a single-copy BAC actuator can result in a significant reduction in growth (Supplementary Fig. 10b).

If the DNAi device has activity in the OFF state, then this could cause selective pressure against its own maintenance. This could lead to loss-of-function mutations that degrade activity over time. In the short-term growth experiments (Fig. 3a), we did not observe a decrease in cell density in the absence of inducer. Still, the effect could be subtle and the device could break when carried over a long period. To test this, cells containing the 3x device, the Y+Z spacer array and the pSC101 target plasmid were passaged in the OFF state for 90 days (Fig. 3b). During this time course, cells were passaged every 12 h into fresh media with selection for the target plasmid (+spectinomycin). Because the cells are maintained under conditions favouring cell growth, this time course involves a remarkable 1,700 cell divisions, corresponding to a 10^520^-fold amplification of the initial inoculant. Every 2 days, a sample of the culture was removed and subjected to the plasmid knockout experiment (Fig. 1c and Methods). Over the entire time course, the plasmids are consistently lost such that they only exist in an average of 2.1 × 10^−8^ of the cell population (Fig. 3b). The stability of target plasmid protein expression levels over the entire course of the experiment was also confirmed by quantifying the RFP signal (Supplementary Fig. 11).

### Programmed cell death by targeting DNAi to the host genome

**Figure 4:**
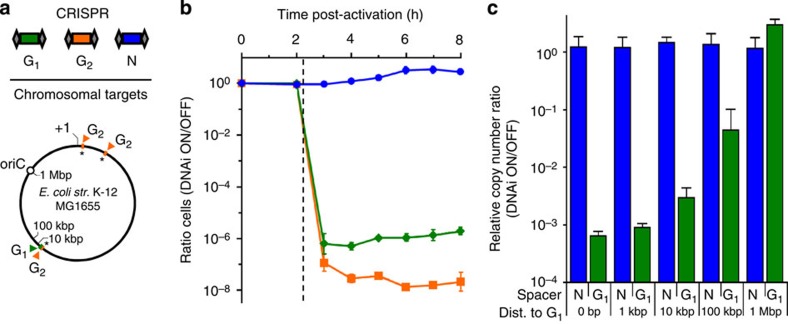
Degradation of specific genomic regions via DNAi and the induction of cell death. (**a**) Two on-target spacers (G_1_ and G_2_) are shown along with the locations of their corresponding sequences in the *E. coli* K-12 host chromosome. The G_2_ spacer targets a site within the chromosomally inserted actuator sequences (starred) and so those proto-spacer locations coincide with the three actuator locations. An off-target spacer (N) is used as a control as it does not target a sequence in the genome. The spacing from the G_1_ target for the qPCR assays is shown. (**b**) The kinetics of cell killing is shown, where the efficiency is presented as a ratio of the titre of viable cells between the DNAi ON (+2 mM arabinose) and OFF (+0.5% glucose) states. The data are shown for the 3x actuator and the G_1_ (pCR-G1) or G_2_ (pCR-G2) targets (green diamonds and orange squares, respectively). The N control (pCR-N) is shown as blue circles. The vertical dashed line indicates *t*=2.25 h, the time at which the P_BAD_ promoter activates to initiate the synthesis of DNAi actuator components (Supplementary Fig. 5a). The corresponding viable cell titre measurements are shown in Supplementary Fig. 12. (**c**) The ability to recover chromosomal DNA sequences via PCR after the induction of DNAi for 8 h is quantified as a function of distance (dist.) from the single chromosomal proto-spacer target. For each chromosomal locus, the efficiency of recovery is measured as the ratio of the relative copy number (RCN) of the DNAi ON state divided by that of the corresponding DNAi OFF state (Methods). Chromosomal distances are measured as the centre-to-centre spacing between the PCR amplicon indicated and the singly occurring G_1_ chromosomal protospacer (NCBI NC_000913.3 position 2,887,466). Sequences for primer pairs are provided in Supplementary Table 5. All data represent the average of three independent experiments performed on different days and error bars indicate the s.d.

The expression of nucleases that degrade the host genome leads to cell death^8^,^40^,^44^,^45^,^50^,^57^,^58^. To determine whether the DNAi device could be used to induce cell death, we constructed host strains containing the 3x device with targeting plasmids encoding a genome-targeted spacer (either G_1_ or G_2_) or an off-target spacer (N, Fig. 4a). The G_2_ spacer targets the attL sequence present in all three of the actuators, which are separated by chromosomal distances of 300 kbp and 2.8 Mbp, respectively. The G_1_ spacer was selected to target only a single chromosomal sequence that is directly adjacent to the actuator at the ΔCRISPR-cas locus. Both the G_1_ and G_2_ spacers are oriented such that their respective crRNAs base pair with the genomic (−) strand upon target recognition.

Strains containing G_1_, G_2_ or N were induced with 2 mM arabinose (DNAi ON) or repressed with 0.5% glucose (DNAi OFF, Fig. 4b). Over an 8-h period, the ratio of viable cells for each spacer was measured as the cell titre of the corresponding strain in the ON state divided by that of the strain in the OFF state (Methods). For both the strains containing G_1_ or G_2_, when the DNAi device is turned ON, cell death is very rapid following a lag phase that is analogous to that observed for the plasmid knockouts (Fig. 1d). The spacer corresponding to multiple sites in the genome is more effective at killing cells, leveling off at a viable cell ratio of 1.9 × 10^−8^, which corresponds to 80 c.f.u. per ml. When the DNAi device is OFF (0.5% glucose), no cell death is observed after 8 h (Supplementary Fig. 12). This is consistent with the long-term plasmid stability that is observed when a targeting spacer is carried but the device is OFF (Fig. 3b). Together, these observations highlight the negligible leakage of the device and its ability to be carried without imparting its activity until induced, even when carrying a toxic genome-targeted spacer.

One advantage of using specific endonucleases is that particular regions of the genome can be targeted for degradation. This could be used to preferentially remove DNA associated with a highly engineered region or insertions containing synthetic pathways. Because the exonuclease activity chews the DNA from either end of the cut, it is expected that there will be less recoverable DNA around the region of the targeted sequence. We applied qPCR to test this using the G_1_ spacer, which is centred on position 2,887,466 of the *E. coli* str. K-12 genome (NCBI numbering), and compared it with the control (N). Samples were induced and qPCR was used to amplify five genomic loci at different distances from the G_1_ cut site (Fig. 4c, Methods). The amount of DNA remaining from the region surrounding the target (0–1 kb) is reduced >10^3^-fold. By 10 kb, there is ∼3.3-fold more DNA, but there is still significant loss. Notably, the DNAi device is carried within 10 kb of each G_2_ spacer. This is a demonstration of the system exhibiting targeted degradation of its own genetics. By 1 Mb, the amount of DNA equals that which is recoverable from the N control. This demonstrates that the specificity of the Cas complex can be applied to selectively target regions of the genome while leaving non-targeted regions intact.

## Discussion

We have demonstrated that the CRISPR machinery can be used to degrade specific intracellular DNA in an inducible and targeted manner. When a plasmid is targeted, it is rapidly lost from a population, and when the genome is targeted, this results in rapid cell death. The DNAi device is non-toxic when carried in the genome, is stable for months and does not impart background activity until induced. This stability does not come at a cost of effectiveness. Indeed, the ability to reduce the number of viable cells by 1.9 × 10^−8^ is among the most effective switches for programmed cell death reported to date^59^ and many of these other systems impart a load on the cell.

An industrial accident could release 10^17^–10^19^ cells into the environment^12^. In such a scenario, the reduction of viable cells by a factor of 1.9 × 10^−8^ is equivalent to reducing the viable cells in a 1 M litre fermenter to those present in 19 ml of culture. This is well below the log 6 reduction in viable cells in the liquid/solid waste stream required by the US Environmental Protection Agency and close to the log 8 reduction required by the US National Institutes of Health (NIH) *E. coli* host-vector (EK2) guidelines^60^ for plasmid retention before disposal. DNAi is also able to degrade plasmid and genomic DNA by 10^8^-fold and the target specificity of the CRISPR machinery allows the nuclease activity to be targeted to recombinant DNA or regions desired to be undetectable. Whether due to accidental release or the use of biomass as fertilizer, the extracellular DNA eventually decays because of nucleases produced by the soil microbiota^11^. Experiments with recombinant *E. coli* showed that DNA could be detected by PCR for 28–60 days after release with half-lives (*τ*_1/2_) of days, with large variation due to soil type and environment^11^,^12^. Beginning with 10^8^-fold less DNA reduces the detection time by ∼27*τ*_1/2_, which can reduce the detection time from months to days.

Historically, biology's capacity to replicate and propagate its DNA has been exploited to procure genetically modified samples without the developer's consent. During the 1950s and 1960s, at the height of phage research, investigators toyed with the idea of obtaining samples of mutant phage variants from competing groups by swabbing the letters and envelopes used for written correspondence between the two labs^61^. In the past, withholding DNA constructs from competitors outright or through a restrictive material transfer agreement was common, but advances in DNA synthesis have made it trivial to bypass these barriers. In a case of industrial espionage, a researcher was convicted in 2011 for the theft of trade secrets from Dow Agrosciences and Cargill and their transfer to competing corporate and academic interests^62^. The theft involved the physical transfer of strains as well as an electronic DNA sequence for a proprietary enzyme, with the associated loss valued at $7 million (USD)^62^.

This manuscript introduces a genetic device that is able to degrade targeted DNA upon the activation of a transcriptional input. For environmental and intellectual property applications, the input could be changed to connect to sensors that activate when the cells are removed from a defined media or exposed to particular conditions (for example, light or oxygen). More sophisticated control can be achieved by connecting the device to the output promoter of a transcriptional genetic circuit; for example, genetic logic to integrate signals from multiple sensors that define a cocktail of chemicals specific to a media or environment^59^,^63^. Similar control could be implemented for applications that make use of the ability of the device to target-specific DNA while leaving the remainder intact. For example, enzymes encoded on a plasmid could be permanently eliminated at a late stage of fermentation. The DNAi device enables these functions while being carried silently in the host genome before activation. Furthermore, the DNAi device is compatible with other systems for the containment of genetically modified organisms, including devices that implement programmed cell death, DNA deletion and the re-coding of the genome to require non-natural amino acids^59^,^64^. One can imagine combining these systems to create layers of redundancy to ensure that engineered organisms are viable in particular environments and if they escape, they eliminate their synthetic DNA.

## Methods

### Strains and media

All strains were derived from a triple-knockout *E. coli* MG1655 Δ(araC-araBAD) ΔPlacI:LacI Δ(cas3-CRISPR) parent (Supplementary Table 2). Standard plasmids were cloned and propagated using an *E. coli* Mach1-T1^R^ host (F^−^ ϕ80(*lac*Z)ΔM15 Δ*lac*X74 *hsd*R(r_K_^−^m_K_^+^) Δ*rec*A1398 *end*A1 *ton*A; Life Technologies), while plasmids containing an R6Kγ origin of replication were cloned and propagated using an *E. coli* TransforMax EC100D *pir*-116 host (*F*^*−*^ *mcrA Δ(mrr-hsdRMS-mcrBC) ϕ80dlacZΔM15 ΔlacX74 recA1 endA1 araD139 Δ(ara, leu)7697 galU galK λ- rpsL (Str*^*R*^*) nupG pir-116(DHFR)*) (Epicenter). Cells were plated on LB (LB Miller Medium, Difco, #244620) supplemented with 1.5% (w/v) agar (Bacto Agar, Difco, #214010), and liquid cultures were grown in 2YT media (2xYT (Yeast Extract Tryptone) Medium, Difco #244020) for all experiments. Ampicillin (100 μg ml^−1^; sodium ampicillin, Gold Biotechnology #A-301-25), kanamycin (50 μg ml^−1^; kanamycin sulfate, Gold Biotechnology #K-120-10), spectinomycin (100 μg ml^−1^; spectinomycin dihydrochloride, Gold Biotechnology #S-140-5) and/or chloramphenicol (35 μg ml^−1^; USB #23600-25G) were used as appropriate. L-arabinose (Ara; Sigma-Aldrich #A3256-100G) or glucose (Glc; BDH #BHD8005-500G) was used as inducer or repressor, respectively.

### Strain construction

Genomic knockouts were performed via the λ_RED_ method using a linear kanamycin-resistant cassette amplified from pKD13 (ref. 65, Supplementary Table 3). Knock-ins of the ∼11 kb DNAi actuator plasmids (pACT-01 and -02, Supplementary Fig. 1b) or their fluorescence measurement plasmids (pGFP-01 and pGFP-02, Supplementary Fig. 1c) were achieved by adapting high-efficiency site-specific chromosomal integrations^66^. First, the *Streptomyces* phage PhiK38−1 *attB* site^67^ (Supplementary Fig. 1a, Supplementary Table 4) was inserted into the host chromosome via the λ_RED_ method. Next, the linked Kan^R^ marker was removed via pCP20 (ref. 65), and the resulting attB^+^ Kan^S^ host was transformed with a temperature-sensitive plasmid (pInt5_ts_, Amp^R^) expressing the cognate phage integrase Int5 under the control of a P_BAD_ promoter^67^ (Supplementary Fig. 1a). Transformants carrying the integrase plasmid were grown in 2YT containing ampicillin and 1 mM arabinose in a shaking incubator at 30 °C and 250 r.p.m. to an OD_600_∼0.5, at which point electrocompetent cells were prepared. The resulting Int5-expressing cells were transformed with an integrative actuator plasmid (pACT-01 or pACT-02, Kan^R^, non-replicative R6Kγ origin) containing the PhiK38–1 *attP* site via electroporation and subsequently recovered in 2YT at 37 °C for 3 h. Recombinants were selected for by growth on LB plates containing kanamycin at 37 °C. Successful chromosomal integration at the targeted locus was confirmed with colony PCR, and loss of the temperature-sensitive Int5 expression plasmid was confirmed by Amp^S^ phenotype. For each modified genomic locus, a corresponding donor strain in a wild-type MG1655 background was created. These antibiotic marker-linked loci were then serially transferred to the final recipient host via P1_vir_ viral transductions^68^. Finally, the genomically integrated FRT-flanked R6Kγ origin and kanamycin resistance marker was removed by expression of FLP recombinase following transformation with temperature-sensitive plasmid pCP20 (Amp^R^, Cm^R^)^65^. Loss of the FRT-flanked region and curing of pCP20 was subsequently confirmed by colony PCR and the presence of Kan^S^, Amp^S^ and Cm^S^ phenotypes. The 1x DNAi device contains the pACT-01 actuator chromosomally integrated at the native CRISPR-cas locus. The 3x DNAi device contains the pACT-01 actuator chromosomally integrated at the LacI locus, and the pACT-02 actuator integrated at the araC-araBAD and CRISPR-cas loci (Supplementary Tables 2 and 3). The 3x GFP strain was constructed identically, except with pGFP-01 or pGFP-02 chromosomally integrated in lieu of pACT-01 or pACT-02, respectively. Each copy of the actuator or GFP plasmid is associated with its own copy of the *araC* gene, which is under control of either its native P_*C*_ promoter (pACT-01) or a constitutive P_J23117_ promoter (pACT-02).

### Preparation of phagemid virion stock solutions

The viral origin of replication (bp 5,505–5,811) was deleted from M13KO7^69^ to create M13Δori (Kan^R^, p15A), a packaging-defective viral helper plasmid that enables the packaging and secretion of phagemids supplied in *trans* but is incapable of infectious self-propagation. MG1655 (F^−^) cells were contransformed with M13Δori and either a pPAM-TC (RFP^−^, Str^R^) or a pPAM-*NNN* (64 PAM library, RFP^−^, Str^R^) target plasmid. Single colonies were selected by growth on kanamycin and spectinomycin, and were used to inoculate 2YT containing the appropriate antibiotics. Samples were grown for 24 h at 37 °C at 250 r.p.m. The cultures were centrifuged at 4,000*g* at 4 °C, and the virion-containing supernatant was washed with chloroform, centrifuged again at 4,000*g*, and the virion-containing aqueous layer was extracted. High levels of RFP expression from pPAM-*NNN*-RFP in combination with M13 viral protein expression are lethal to the host, and so these plasmids could not be similarly packaged in this manner.

### Plate-based plasmid knockout assay

Host cells containing a DNAi device and an RFP^+^ target plasmid (Str^R^) were transformed with a CRISPR targeting plasmid (Cm^R^), plated onto LB containing all appropriate antibiotics in addition to 0.5% glucose and grown for 12 h at 37 °C. Single colonies were used to inoculate 2YT (1 ml) containing all appropriate antibiotics and 0.5% glucose, and the resulting liquid cultures were grown in a shaking incubator for 3 h at 37 °C and 250 r.p.m. until OD_600_=0.25–0.75. Cultures were spun down at 21,000*g*, washed once with fresh 2YT (1 ml) and back-diluted to OD_600_=0.01 into 2YT (2 ml) containing appropriate antibiotics but without selection for the target plasmid (−spectinomycin). The diluted culture was then split into two 1 ml samples, and DNAi activity was either induced by adding 2 mM arabinose or repressed by adding 0.5% glucose. Cultures were then grown in a shaking incubator for 8 h at 37 °C and 250 r.p.m. and periodically sampled. The fraction of host cells retaining the target plasmid (Target^+^) was calculated from the ratio of colony forming units obtained from plating onto both target-selective (+spectinomycin) and non-selective (−spectinomycin) LB plates containing all other appropriate antibiotics. In our hands, the plate-based assay was imprecise for measuring samples expected to contain a high fraction (>50%) of target-positive host cells (that is, DNAi OFF or off-target CRISPR samples). A cytometry-based assay was used for precise quantification of these samples (see *Cytometry-based plasmid knockout assay (RFP)* below).

### Cytometry-based plasmid knockout assay

Samples were prepared and induced identically to the plate-based knockout assay described above, except that for sampling, aliquots were back-diluted 1:1,000 into 2YT containing 0.5% Glc and all appropriate antibiotics except spectinomycin, and then outgrown in a shaking incubator at 37 °C and 250 r.p.m. for 8–12 h. This served to arrest further target plasmid loss while allowing for the dilution of accumulated intracellular RFP in the target-negative cells present. Following outgrowth, aliquots were diluted into PBS containing 1 mg ml^−1^ kanamycin to arrest further growth and protein synthesis and stored at 4 °C. Cells were analysed using a BD Biosciences LSRFortessa. The fraction of cells containing the target plasmid was calculated as fraction of cells with red fluorescence values greater than a 1,000 arbitrary units cut-off. Data were analysed using FlowJo (TreeStar Inc., Ashland, OR), and populations were gated on the basis of forward and side scatter. For all samples, the gated population contained between 10^4^ and 10^5^ cells.

### Plasmid knockout kinetic assay (PAM experiments)

Samples were prepared as for the plate-based knockout assay, except the sample volumes were reduced to 500 μl and all cultures were grown in 96-well format in a shaking incubator at 37 °C and 990 r.p.m. Cultures were sampled and subsequently outgrown as described for the cytometry-based knockout assay. For targets with strong PAM sequences (AAA, AAC, AAG, AAT, AGG, ATG, GAG or TAG), experiments were carried out for 4.5 h with sampling every 10 min beginning at 2 h post induction. For targets with slow (CAG, GGG, GTG, TAA or TAG) or intermediate (ATA or TTG) PAM sequences, experiments were carried out for 8 h with sampling every 15 min starting at 3 h post induction. Following outgrowth, aliquots were diluted into PBS containing 1 mg ml^−1^ kanamycin to arrest further growth and protein synthesis and stored at 4 °C. Cells were analysed using a BD Biosciences LSRFortessa. Data were analysed using FlowJo (TreeStar Inc., Ashland, OR) and populations were gated on the basis of forward and side scatter. For all samples, the gated population contained between 10^4^ and 10^6^ cells.

### Isolation of target plasmid DNA

Following a plasmid knockout experiment, 500 μl of the resulting culture was collected and spun down at 21,000*g*, and the supernatant was removed and discarded. Any resulting pellets were stored at −20 °C until isolation could be performed in parallel. For plasmid knockout samples, plasmid DNA extraction was performed using a miniprep kit (Qiagen) and all samples were normalized to a final volume of 100 μl of EB (Qiagen) for subsequent qPCR analysis. On the basis of experiments in which comparable samples of target-negative cells were doped with known amounts of gel-purified target plasmid (pTAR(S)) and then subjected to analogous DNA extraction techniques, the efficiency of miniprep isolation was estimated at 35±18%.

### Isolation of target chromosomal DNA

A 250-μl aliquot of the culture was collected and spun down at 21,000*g*, and the supernatant was removed and discarded. Any resulting pellets were stored at −20 °C until isolation could be performed in parallel. Total genomic DNA was extracted using a Wizard Genomic DNA Purification Kit (Promega) in accordance with the manufacturer's protocol, and all the samples were resuspended in a final volume of 100 μl of TE buffer (Promega) for subsequent qPCR analysis. The efficiency of total genomic DNA isolation was calculated to be 14±3.0% by generating qPCR standard curves from known quantities of target DNA and assuming one copy of the genome per c.f.u. and 3 × 10^9^ c.f.u. per 250 μl sample.

### Quantitative PCR

Quantitative PCR reactions (10 μl total) containing 1 μl of either sample or standard DNA template were prepared from SsoFast EvaGreen Supermix with Low ROX (Bio-Rad) in accordance with the manufacturer's specifications. The samples were amplified and measured using a Mastercycler RealPlex^2^ (Eppendorf) real-time PCR thermocycler. The thermocycling protocol (40 cycles) was as follows: (1) initial denaturation—2:00 at 95 °C; (2) melting—0:03 at 95 °C; (3) annealing/synthesis: 0:30 at 65 °C. Primers and amplicons are described in Supplementary Table 5, and all primers amplified their targets with 100±5% efficiency. For each amplicon, a series of 5-fold serial dilutions was prepared from a 1 ng μl^−1^ stock solution of gel-purified target DNA. Each of the eight dilutions spanning 5^−1^ to 5^−8^ ng μl^−1^ was then subjected to measurement by qPCR in triplicate, and the resulting Ct values were used to prepare a calibration curve of Ct versus relative copy number (RCN), for which the most dilute solution (5^−8^ ng μl^−1^) was arbitrarily designated as RCN=1. When absolute quantification was required, a sample's RCN value was determined using the calibration curve and subsequently converted to an absolute copy number (ACN_sample_; given in copies per μl sample) value using the formula ACN=RCN × (6.02 × 10^23^ copies mol^−1^) × (5^−8^ ng μl^−1^ sample)/M, where M is the molecular weight of the specific dsDNA amplicon (in ng mol^−1^, Supplementary Table 5). The isolated DNA samples loaded into the qPCR reactions were more concentrated relative to the initial cultures from which they were obtained, and so ACN_sample_ was then converted to ACN_culture_ (given in copies per ml culture) according to the formula ACN_culture_=ACN_sample_ × (1,000 μl ml^−1^)/C, where C is equal to the initial volume of culture sampled divided by the final volume of the isolated DNA sample. For miniprepped DNA (500 μl culture into 100 μl EB) C=5, and for isolated genomic DNA (250 μl culture into 100 μl TE) C=2.5.

### Measuring the impact on cell growth

Strains containing variants of the DNAi devices were co-transformed with both a target plasmid and a corresponding on-target CRISPR plasmid, plated onto LB containing antibiotics in addition to 0.5% glucose, and grown for 12 h at 37 °C. Single colonies were used to inoculate 2YT (1 ml) containing all appropriate antibiotics and 0.5% Glc, and the resulting liquid cultures were grown in a shaking incubator for 3 h at 37 °C and 250 r.p.m. until OD_600_=0.25–0.75. Cultures were spun down at 21,000*g*, washed once with fresh 2YT (1 ml), diluted to OD_600_=0.01 into 2YT (3 ml) containing kanamycin and/or chloramphenicol as appropriate and then split into 3 × 1 ml samples in a 96-well format. Each sample was induced with Ara (0, 2 or 10 mM) and then grown in a shaking incubator for 8 h at 37 °C and 250 r.p.m. The viable cell titre of each sample was then measured by plating serial dilutions onto non-selective (−spectinomycin) LB plates containing all other appropriate antibiotics.

### Evolutionary stability experiments

The strain containing the 3x DNAi device was transformed with the on-target Y+Z dual CRISPR plasmid (pCR-YZ, Cm^R^) and a pSC101 target plasmid (pTAR(S), RFP^+^, Str^R^), plated onto LB containing chloramphenicol and spectinomycin in addition to 0.5% Glc, and grown for 12 h at 37 °C. A single colony was used to inoculate 2YT (1 ml) containing chloramphenicol, spectinomycin and 0.5% Glc (‘cell passage media'), and the resulting liquid culture was grown for 3 h in a shaking incubator at 37 °C and 250 r.p.m. until OD_600_=0.25–0.75. These cells were washed with 2YT (1 ml), and then back-diluted to OD_600_=10^−5^ into fresh cell passage media (1 ml) to mark the start of the experiment (‘day 1', *t*=0). The culture was grown continuously in a shaking incubator at 37 °C and 250 r.p.m. with back-dilution to OD_600_=10^−5^ (75,000- to 100,000-fold) into fresh cell passage media (1 ml) occurring every 12±2 h. In parallel with every fourth such back-dilution (corresponding to a ∼48-h period) beginning with the first occurring at *t*=0 measurements of DNAi device knockout efficiency and target plasmid stability were performed on a sample removed from the passaged culture in accordance with aforementioned protocols (see both ‘*Plasmid knockout assay*' sections above). Continuous culture and periodic measurement of the sample were performed in this manner for a period of 90 days.

### Cell death assays

The strain containing the 3x DNAi device was transformed with either an on-target (pCR-G1 or -G2, Cm^R^) or an off-target CRISPR targeting plasmid (pCR-N_2_, Cm^R^), plated onto LB containing chloramphenicol in addition to 0.5% Glc, and grown for 12 h at 37 °C. Single colonies were used to inoculate 2YT (1 ml) containing chloramphenicol and 0.5% glucose, and the resulting liquid cultures were grown in a shaking incubator for 3 h at 37 °C and 250 r.p.m. until OD_600_=0.25–0.75. Cultures were spun down at 21,000*g*, washed once with fresh 2YT (1 ml) and back-diluted to OD_600_=0.01 into 2YT (2 ml) containing chloramphenicol. The diluted culture was then split into two 1-ml samples, and DNAi activity was either induced by adding 2 mM arabinose (DNAi ON) or repressed by adding 0.5% glucose (DNAi OFF). Cultures were then grown in a shaking incubator for 8 h at 37 °C and 250 r.p.m. with periodic removal of samples for analysis. For each sample, the titre of viable cells (in c.f.u. per ml) was then determined by 10-fold serial dilution and subsequent plating onto LB agar containing chloramphenicol. The fraction of viable cells was then calculated as the ratio of the cell titre in the DNAi ON state to that in the DNAi OFF state for a given CRISPR target.

## Additional information

**How to cite this article:** Caliando, B. J. *et al*. Targeted DNA degradation using a CRISPR device stably carried in the host genome. *Nat. Commun.* 6:6989 doi: 10.1038/ncomms7989 (2015).

## Supplementary Material

Supplementary Information Supplementary Figures 1-12 and Supplementary Tables 1-8
