# Supplementary material for: Targeted DNA degradation using a CRISPR device stably carried in the host genome
Source: Nat Commun. 2015 May 19;6:6989. doi: 10.1038/ncomms7989 (PMC4479009; doi:10.1038/ncomms7989)
Supplement: Supplementary Information — Supplementary Figures 1-12 and Supplementary Tables 1-8 [file ncomms7989-s1.pdf]

Supplementary Figure 1

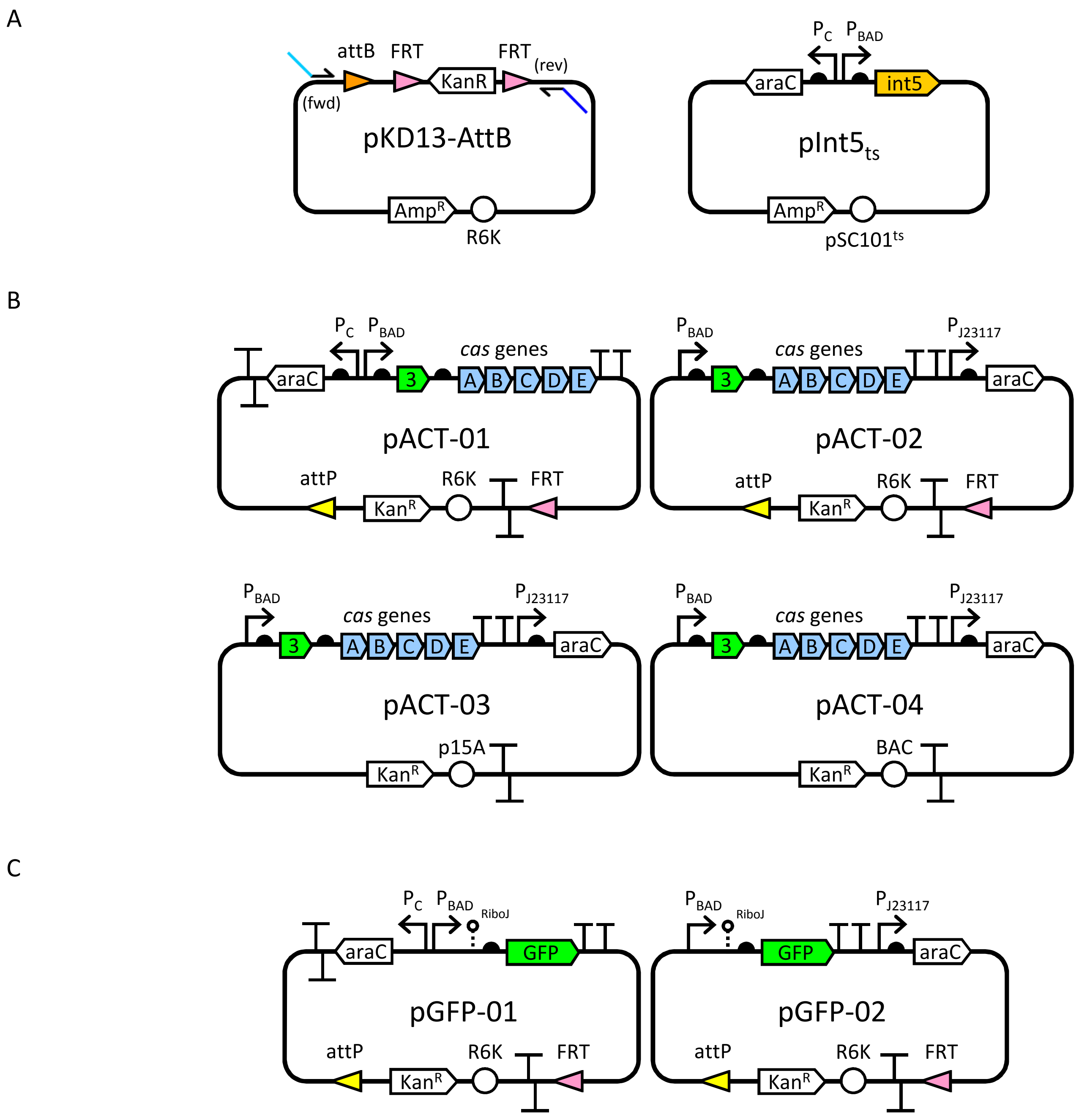

<u>Supplementary Figure 1</u>: Strain construction, actuator, and fluorescence measurement plasmid maps.
(A) pKD13-attB encodes a modified Kan^R knockout cassette coupled to an Int5 attB site. Upon amplification with appropriate primers ("fwd" and "rev") encoding 5'- and 3'- homology (light and dark blue overhangs, respectively) to a target site within the host genome, the resulting linear DNA can be transformed into a λ_{RED}-expressing host to both knockout the targeted region and knock in a chromosomal attB site. pInt5ts confers arabinose-inducible expression of the Int5 site-specific recombinase. The $pInt5_{ts}$ plasmid possesses a temperature-sensitive pSC101 origin of replication that allows the plasmids to be cured from the host via growth at 37°C following successful recombination. **(B)** For all actuator plasmids, expression of the *cas3ABCDE* operon is under the control of the arabinose-inducible P_{BAD} promoter. P_{BAD} function requires the expression of the AraC transactivator protein, and actuator plasmids assume two different organizations with respect to *araC*. Either the *araC* gene is expressed under the control of its native, auto-regulated P_C promoter (pACT-01), or it is under weak constitutive expression from a PJ23117 promoter (pACT-02 through pACT-04). The attP sites present in pACT-01 and pACT-02 enable Int5-mediated recombination with a corresponding chromosomal attB site thereby allowing these plasmids to integrate into the host chromosome via loop-in mechanism. Both pACT-01 and pACT-02 possess R6K origins in order to ensure that in a *pir*- host such as *E. coli* MG1655, unregulated replication of chromosomal DNA does not initiate from these loci. The FRT site present in pACT-01 and pACT-02 allows the plasmid Kan^R marker and R6K origin to be excised via FLP-mediated recombination between the plasmid-borne FRT site and an adjacent chromosomal FRT site. **(C)** Fluorescence measurement plasmids pGFP-01 and pGFP-02 are isogenic with actuator plasmids pACT-01 and pACT-02, except that a RiboJ-B0034-GFP reporter cassette was substituted for the actuator *cas* operon. The were integrated instead their corresponding pACT counterparts at the same three chromosomal locations to create the 3x GFP reporter strain (Methods).

Supplementary Figure 2

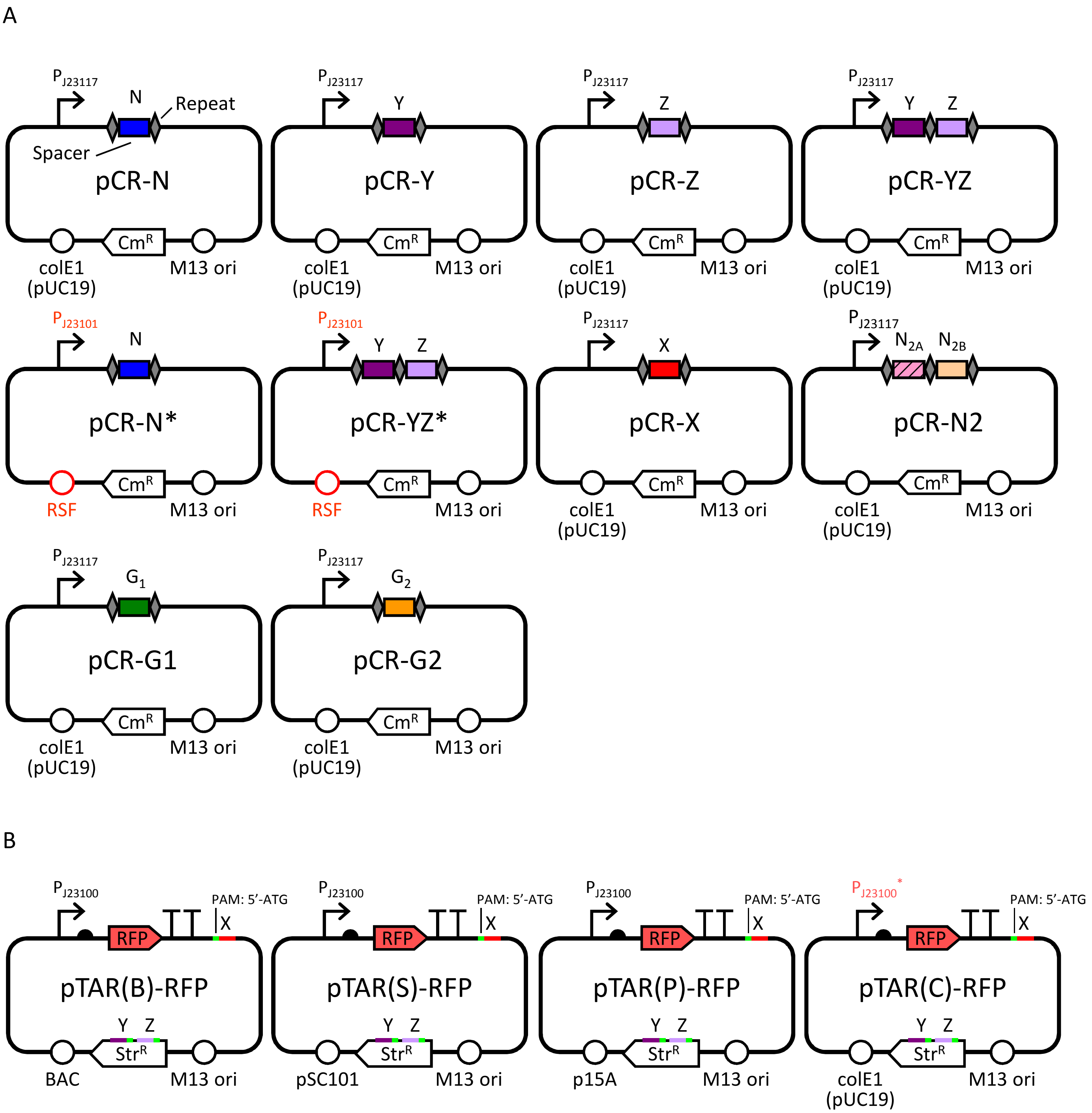

<u>Supplementary Figure 2</u>: CRISPR targeting plasmid and basic target plasmid maps.
(A) All DNAi device experiments require at least one repeat-flanked CRISPR spacer to direct DNAi activity. The transcription of all CRISPR arrays was constitutive. Due to the high copy number of the pUC19-based colE1 plasmid, a weak P_{J23117} promoter was used. When experimental conditions created a conflict between the CRISPR plasmid's origin of replication and that of the target plasmid (Figs 1D and 1E), the CRISPR plasmid colE1 origin was substituted with an RSF origin (highlighted in red). For these RSF variants, a stronger constitutive promoter (P_{J23101} – highlighted in red) was used in order to offset the drop in crRNA expression due to lower CRISPR plasmid copy number. **(B)** The RFP-expressing targets used for all plasmid knockout experiments are homologous and span more than two orders of magnitude with respect to copy number (1-2 copies/genome for BAC up to >500 copies/genome for pUC19). All copy number variants constitutively express high levels of RFP, confer resistance to streptomycin (and spectinomycin), and possess a non-coding proto-spacer (X) coupled to an ATG PAM in addition to two StrR-coding proto-spacers, Y and Z, with ATG and AAG PAMs, respectively. Increasing target plasmid copy number with a pUC19-based colE1 origin caused RFP-mediated toxicity when expressed from the strong promoter $P_{J23100.}$ An attenuated promoter (P_{J23100}*) in which the -10 element had been mutated from TACAGT to TACTAT was used instead.

Supplementary Figure 3

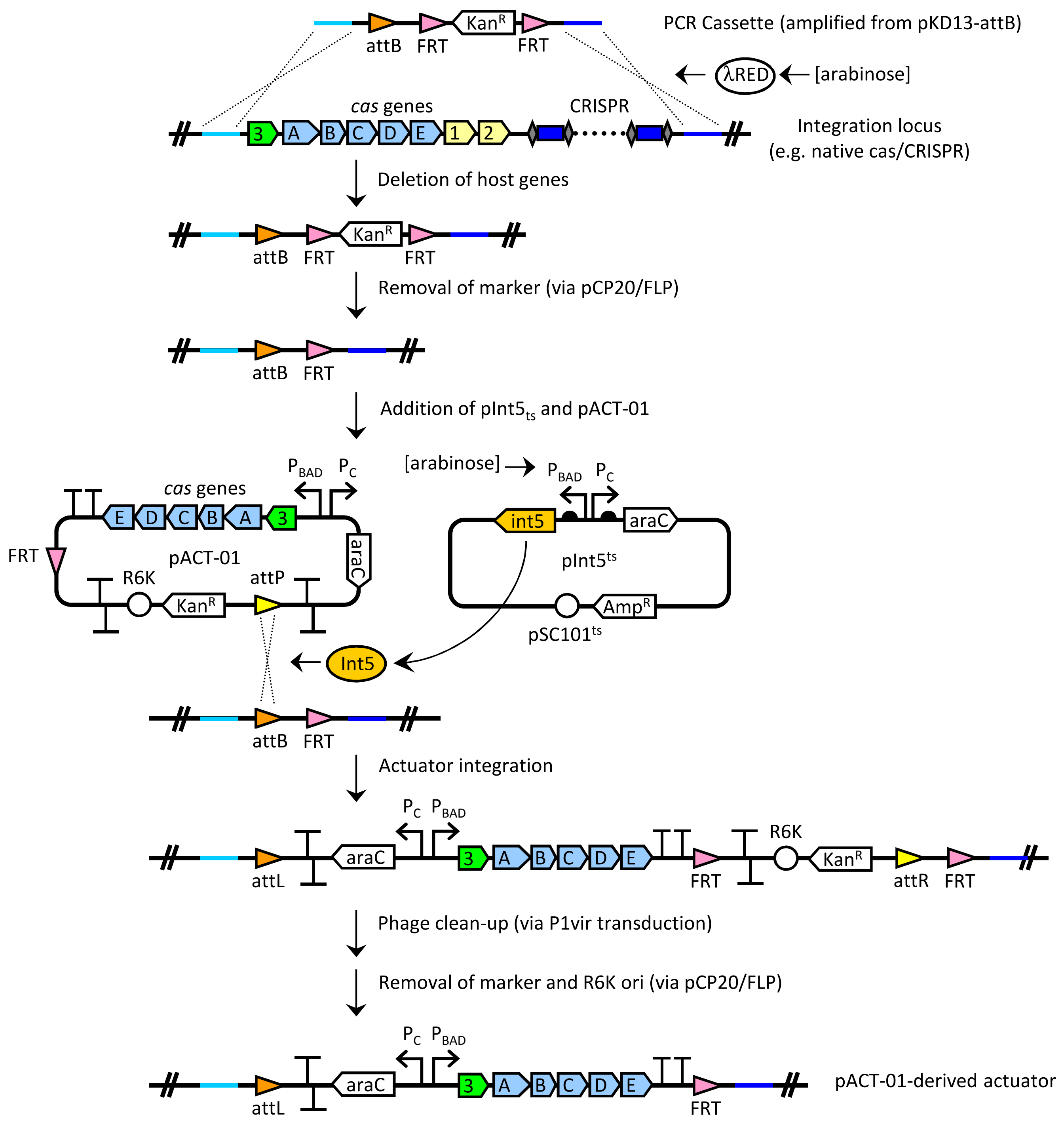

Supplementary Figure 3: Schematic of actuator genomic integration methodology.
A Kan^R knockout cassette containing the Int5 attB site is amplified from pKD13-attB using primers containing 5'- and 3'-homology (light and dark blue segments, respectively) to regions of the MG1655 chromosome targeted for actuator insertion (See Supplementary Table 3 for primer sequences). The attB cassette is integrated into an MG1655 donor strain via λ_{RED} recombinase[91], thereby simultaneously deleting a segment of the host chromosome. (See Supplementary Table 2 for deleted segments) The Kan^R marker is removed via FLP recombinase (pCP20)[91], and the marker-free strain is transformed with pInt5$_{ts}$. Int5 expression is induced with arabinose and transformed with an integrating actuator plasmid (i.e. pACT-01 as shown). Int5-mediated recombination between the plasmid-borne attP site and the chromosomal attB site then creates a chromosomally-integrated actuator sequence within the donor strain. The integrated actuator locus is then moved from the donor strain to the final recipient host strain via P1$_{vir}$ phage transduction. The Kan^R marker, R6K origin, and Int5 attR site are subsequently removed from the recipient host via FLP recombinase (pCP20) to yield the final 1x actuator strain. For host strains containing the 3x genomic actuator, an analogous procedure was iterated two additional times using pACT-02 as the actuator.

Supplementary Figure 4

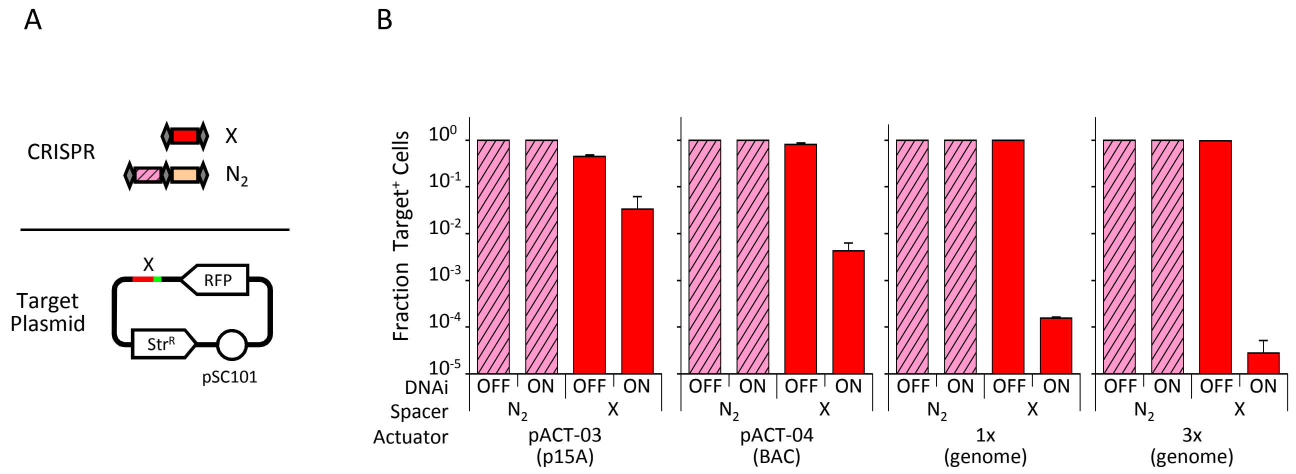

Supplementary Figure 4: Characterization of the DNAi device carried on different plasmid backbones and genomic configurations.
(A) The designs for target spacer sequences are shown. X is present in a non-coding region of the pSC101 target plasmid (pTAR(S)) with an ATG PAM, and neither of the dual N_2 spacers correspond to a valid proto-spacer target in either the plasmid or the host genome. Sequences for these spacers are provided in Supplementary Table 1 **(B)** Data for the knockout of target plasmid are shown for different spacers in combination with different actuators (genomic 1x and 3x, and plasmid-borne p15A and BAC ori). The fraction of cells that retain the pSC101 origin pTAR(S) target plasmid (Target$^+$) is shown in the OFF (repressed by 0.5% glucose) and ON (2mM arabinose) states. The OFF data and the 1x/N ON data were obtained using the cytometry assay and the remainder were obtained by the plate-based assay (Methods) . All data represent the average of three independent experiments performed on different days and the error bars are the standard deviation.

Supplementary Figure 5

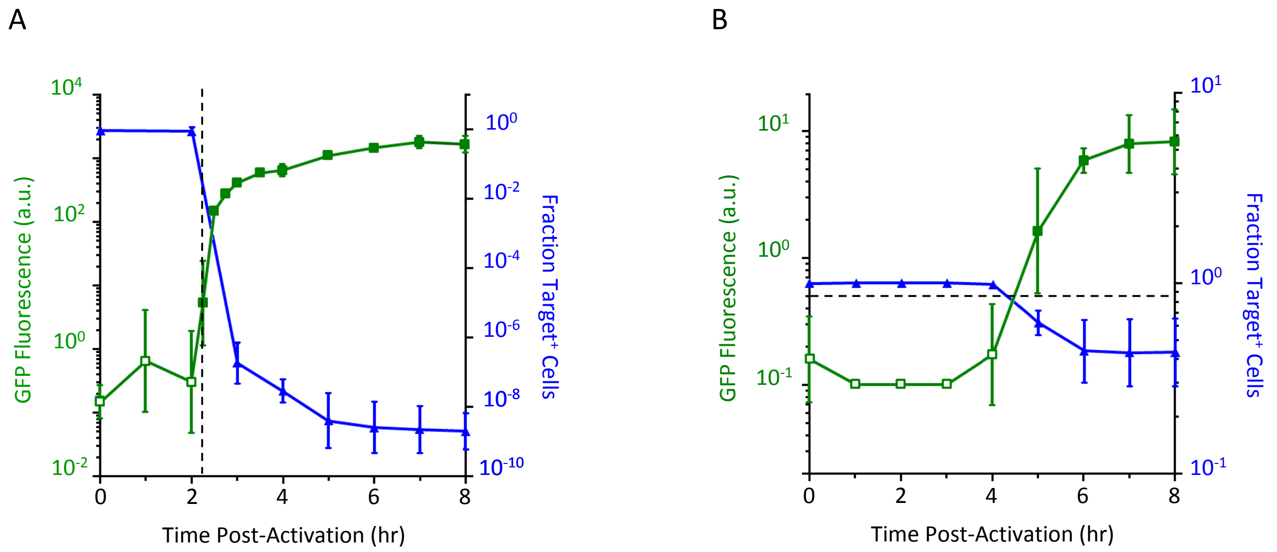

<u>Supplementary Figure 5</u>: Characterization of P_{BAD} Input

(A) P_{BAD} input promoter expression levels over time for the 3x GFP reporter strain (green squares, left-hand axis in a.u., Methods) are shown for cells grown under standard DNAi induction conditions (2YT, 2mM Ara). The data from Fig. 1D depicting the kinetics of pTAR(S) plasmid knockout using the 3x DNAi strain containing pCR-YZ (blue triangles, right-hand axis in fraction target+ cells) are overlaid for reference. Cultures of the 3x GFP strain containing pTAR(S) and pCR-YZ were prepared, induced, cultured, and sampled in a manner identical to that used in Fig 1D (Methods). Cytometric analysis of GFP fluorescence was also performed similarly, except that populations were gated based on forward and side scatter and then based on RFP expression from pTAR(S) (1000 RFP au cut-off) to ensure that only viable cells were counted. The vertical dashed line at t = 2.25 hr indicates the maximal lag time for P_{BAD}-mediated synthesis of the GFP reporter. This lag correlates exceptionally well with the observed lag in the induction of DNAi-mediated plasmid knockout. **(B)** P_{BAD} input promoter expression levels over time for the 3x GFP strain (green squares, left-hand axis) and the kinetics of pTAR(S) plasmid knockout for the 3x DNAi strain (blue triangles, right-hand axis) are shown for cells grown under leakage conditions (2YT, 0mM Ara, 0% Glc). Again, both strains contained the pTAR(S) & pCR-YZ target/CRISPR plasmid pair. With the exception of the inducer concentrations, experiments were performed identically to those depicted in (A). The horizontal dashed line at 5×10^{-1} a.u. reflects the estimated minimum expression threshold for DNAi activity, defined as the expression level required to observe a fraction of target-positive cells <95% in the corresponding plasmid knockout experiment, and is approximately ~3-fold higher than the expression level of P_{BAD} in the glucose-repressed state (corresponding to t = 0 hr, Methods). All measurements were background-corrected by subtracting the auto-fluorescence of a triple-knockout *E. coli* MG1655 parent strain (No DNAi, Methods) containing target and CRISPR plasmids pTAR(S) and pCR-YZ that was grown under identical conditions. Data points depicted as hollow green squares include measurements whose value was at or below the detection limit for the instrumentation used, and were therefore rounded up to the minimum measurable value (0.1 a.u.). All data represent the average of three independent experiments performed on different days, and error bars indicate the standard deviation.

Supplementary Figure 6

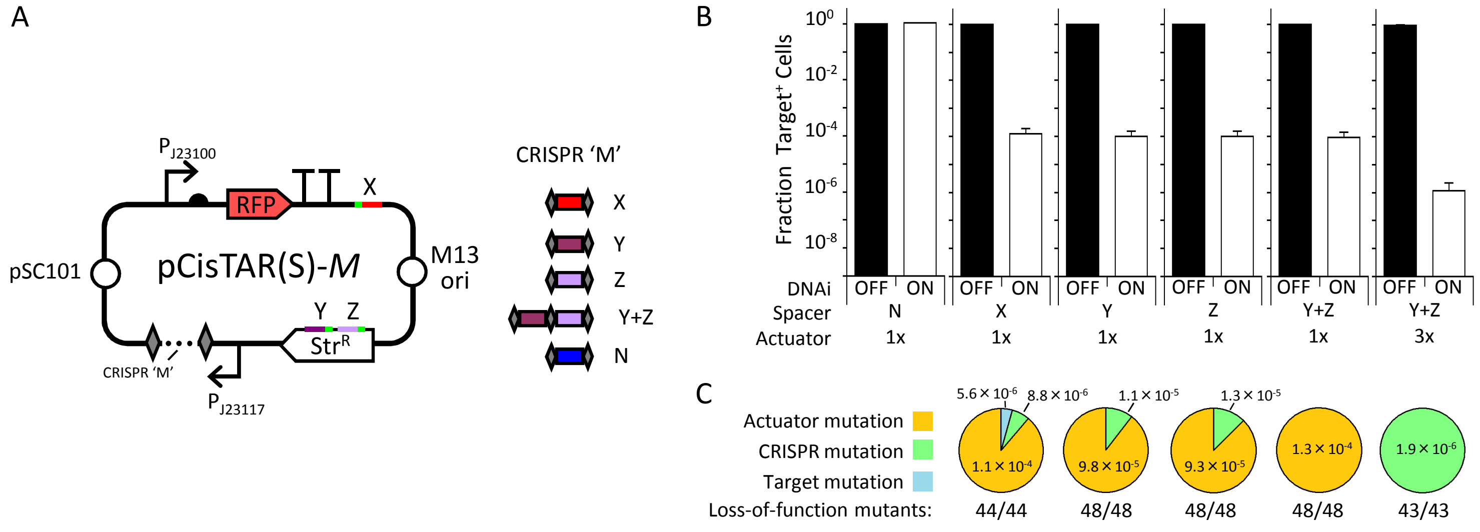

<u>Supplementary Figure 6</u>: Characterization of DNAi device functionality and escape mutants.
(A) The map for the pCisTAR(S)-*M* set of *cis*-targeting plasmids, which encode both a constitutively-expressed spacer ('*M*') and its corresponding proto-spacer, is depicted. The set is otherwise isogenic with pTAR(S). Spacer combinations are shown on the right, and their sequences, along with the PAMs associated with each of their respective proto-spacers, are provided in Supplementary Table 1. **(B)** Data for the knockout of *cis*-targeted plasmid are depicted for different spacers in combination with different actuators (genomic 1x and 3x). The fraction of cells that retain the *cis*-targeted plasmid (Target$^+$) is shown in the DNAi OFF (repressed by 0.5% glucose) and ON (2mM arabinose) states. The OFF data and the 1x/N ON data were obtained using the cytometry assay and the remainder were obtained by the plate-based assay (Methods). The ~1000-fold difference in knockout efficiencies between the *cis*-targeted 3x/Y+Z strain depicted above (Target$^+$ fraction = 1.9×10^{-6}) and the *trans*-only targeted 3x/Y+Z strain from Figure 1c (Target$^+$ fraction = 2.1×10^{-9}) stems from the lower redundancy of the CRISPR array in the former case (10-12 pSC101-borne copies vs. >500 pUC19-borne copies). When a 3x DNAi strain harboring both *cis*-targeted pCis-TAR(S)-YZ and *trans*-targeting pCR-YZ plasmids (3x/*cis-trans*Y+Z) was constructed, the overall knockout efficiency in the DNAi ON state increased to a comparable value as expected (Target$^+$ fraction = 2.0×10^{-9}). The DNAi OFF states were also similar (97% vs. >98% retention). All data represent the average of three independent experiments performed on different days and the error bars are the standard deviation. **(C)** DNAi device failure modes for each on-target strain subjected to DNAi ON conditions in (B) are shown. From the +spectinomycin plates corresponding to each strain's DNAi ON samples, 43-48 surviving Str^R colonies (taken from three independent knockout experiments) were selected to innoculate 5-ml cultures of 2YT containing 0.5% glucose and appropriate antibiotics for target selection (+spectinomycin). Cultures were grown at 37°C with shaking at 250 rpm until OD_{600} 0.3-0.8, and then prepared as chemically competent cells using a Mix & Go *E. coli* Transformation Kit (Zymogen) in accordance with the manufacturer's instructions. The resulting 500 µl stock of competent cells from each surviving clone was split into 5x100 µl aliquots in a 96-well format, and each aliquot was then transformed with either an additional pCR targeting plasmid complement (pCR-X, -Y, -Z, or -N, pUC19, Cm^R) or an actuator plasmid complement (pACT-03, p15a, Kan^R) in accordance with the kit manufacturer's instructions. The transformant cell mixtures were recovered in 1 ml 2YT without antibiotics at for 1 hr at 37°C and 990 rpm and then back-diluted 1:200 into 1 ml fresh 2YT containing antibiotics necessary for pCR/pACT-03 selection (+chloramphenicol or +kanamycin). The resulting polyclonal mixtures of transformants were grown overnight at 37°C and 990 rpm, back-diluted 1:1000 (OD ~0.01), and subjected to an additional 8 hr of DNAi inducing conditions (2YT with 2mM Ara, -spectinomycin), at which point the fraction of target$^+$ cells was measure in accordance standard cytometry-based protocols (Methods). The particular loss-of-function mutation class was then assigned based upon which complementation plasmids did or did not restore a surviving clone's DNAi activity (90% target plasmid retention cutoff) using Supplementary Table 8. For all strains depicted in (B), none of the original surviving clones were DNAi$^+$ transient escapees. Instead, 100% of the clones possessed a loss-of-function mutation in either their respective actuator (yellow), CRISPR spacer (green), or proto-spacer target (blue) sequences that rendered the DNAi device inactive. In contrast, when similar analyses were performed on the survivors from knockout experiments with either the 3x/*cis-trans*Y+Z strain or the *trans*-only 3x/Y+Z strain from Figure 1c, none of the 48 clones tested possessed a loss-of-function mutation (not shown). Instead, all survivors were transient escapees, which demonstrated DNAi-mediated plasmid knockout activity upon re-induction (Methods). Thus, if the probability of each component's failure is sufficiently minimized, then the DNAi device's knockout efficiency becomes kinetically-limited rather than mutation-limited. The numbers labeling each pie sector indicate the predicted fraction of total cells from the experiment in (B) that contain the specified mutation, and this fraction was calculated as [Fraction target$^+$ DNAi ON cells from (B)] × [# Clones containing specified mutation]/[Total # clones analyzed]. Pie charts are vertically aligned with the corresponding strain in (B).

Supplementary Figure 7

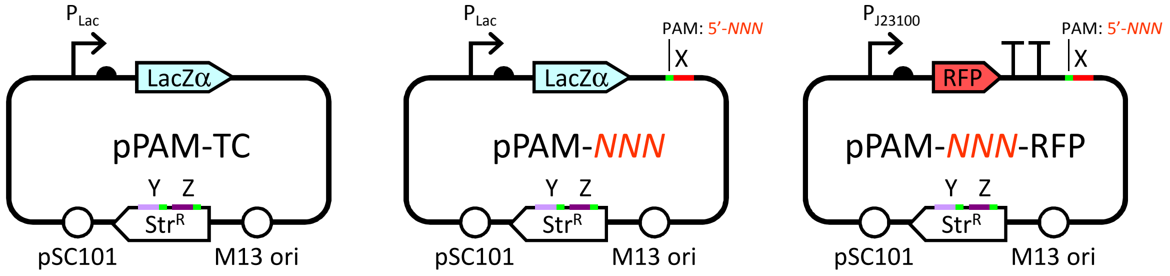

<u>Supplementary Figure 7</u>: Maps of target plasmids used in PAM experiments.
All PAM targets possess a pSC101 origin of replication. The additional M13 origin of replication allows these plasmids to be packaged as ssDNA into a viral capsid when the host co-expresses the full compliment of M13 viral proteins. The resulting plasmid-containing phagemid particles are capable of directly transducing F^+ *E. coli* strains.

Supplementary Figure 8

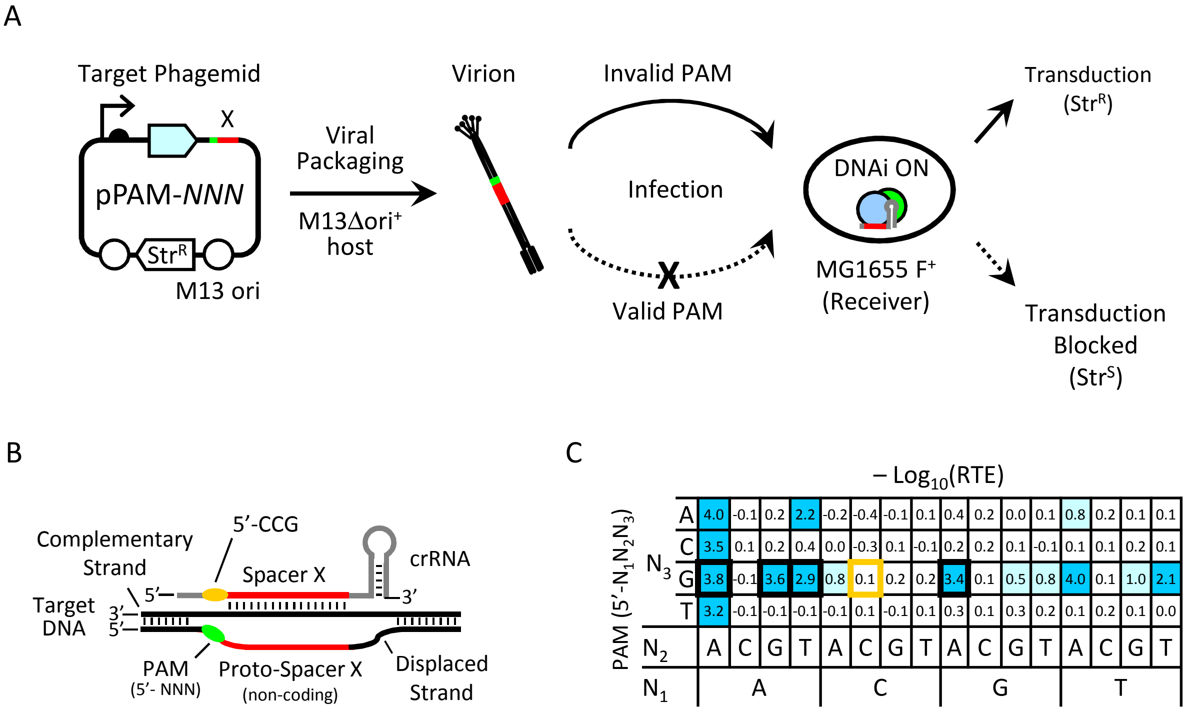

Supplementary Figure 8: Active PAM identification and classification via phagemid transduction assay

(A) Schematic of M13-based phagemid transduction blocking assay. Host receiver cells (MG1655 F^{+}) were co-transformed with a p15A-based actuator plasmid (pACT-03, Kan^{R}) and either an on-target (pCR-X, Cm^{R}) or off-target (pCR-N2, Cm^{R}) CRISPR targeting plasmid. single colonies were selected by growth on kanamycin and chloramphenicol plates and were used to inoculate 2YT containing the appropriate antibiotics. Samples were grown overnight in a shaking incubator at 37°C and 250 rpm, back-diluted to OD_{600} ~0.05 into fresh 2YT containing antibiotics, induced with 2mM Ara, and then grown for an additional ~3-4 h at 37°C and 250 rpm until OD_{600} 0.3-0.7. Cultures were concentrated 50-fold and split into 10 µl aliquots. To each induced aliquot, 10 µl of target plasmid phagemid (pPAM-*NNN*, Str^{R}) stock solution (10-fold dilution, Methods) was added. Infections were incubated at room temperature for 5 min, diluted with 80 µl of additional 2YT, and recovered at 37°C for 1 hr without shaking. The titer of target-positive transductants (in cfu/µl) was then measured by plating 10-fold serial dilutions of each infection onto LB containing spectinomycin. The raw relative transduction efficiency (RTE_{RAW}) for each PAM phagemid in the library was then calculated as (# transductants X strain)/(# transductants N_2 strain). This raw value incorporates both CRISPR-dependent and CRISPR-independent differences in transduction efficiency between the on-target (X) and off-target (N_2) strains. Therefore, in order to control for any possible CRISPR-independent differences in transduction, infection with a control phagemid (pPAM-TC), which entirely lacked proto-spacer X, was similarly quantified for both strains. The final, corrected relative transduction efficiency (RTE) was then calculated as RTE = K x RTE_{RAW}, where K is the CRISPR-independent correction factor equal to (# transductants N_2 strain)$_{TC}$/(# transductants X strain)$_{TC}$ for the pPAM-TC transductions. **(B)** Schematic highlighting the role of the model R-loop formed upon Cascade binding to non-coding proto-spacer X (protein components omitted for clarity)[41,43,49,50]. **(C)** The effect of PAM sequence on phagemid transduction efficiency. Data reflect the negative log-scale RTE values for each PAM as determined via the phagemid blocking assay described in (A). Ten strong PAMs (dark blue; -Log(RTE) > 2.0) and 5 weak PAMs (light blue; 0.5 < -Log(RTE) < 2.0) were identified in this manner. The previously identified canonical PAM sequences are outlined in black[49]. The gold outline corresponds to 5'-CCG, which matches the 3 nt of the crRNA immediately 5' to the spacer sequence as depicted in (B) and was used as a negative control.

Supplementary Figure 9

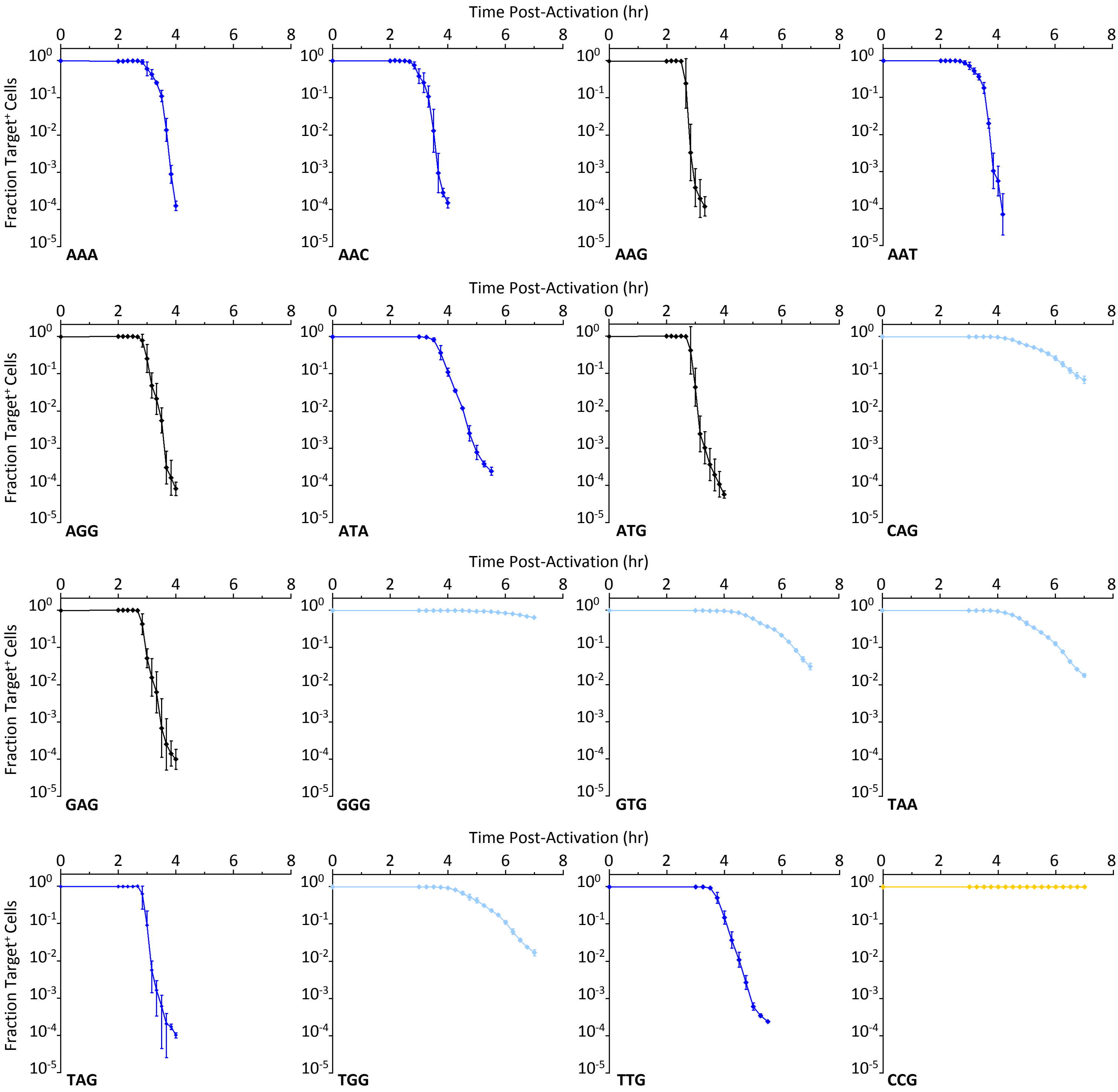

<u>Supplementary Figure 9</u>: The dynamics of plasmid loss for each active PAM sequence.
eviation. The overlay from Figure 2b is separated into individual plots for increased visual clarity. PAM sequences are indicated in the lower left of each graph, and colors correspond canoncial, strong, weak, and negative control PAMs as in Supplementary Figure 8. Data are for the 1x DNAi device, X spacer targeting plasmid (pCR-X, CmR), and a pSC101 target (pPAM-NNN-RFP, RFP+, StrR) and reflect the fraction of cells containing the target plasmid as determined by PAM kinetic assay (Methods). All data reflect the average of three independent experiments performed on different days, and error bars indicate the standard d

Supplementary Figure 10

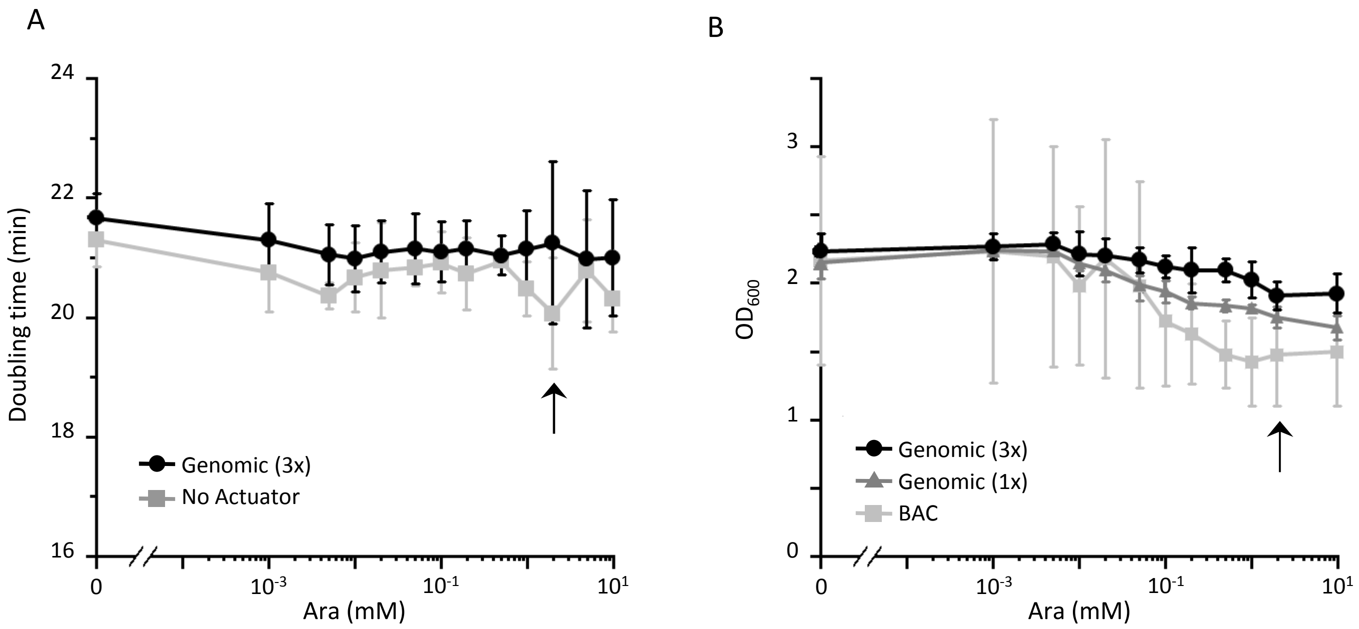

<u>Supplementary Figure 10</u>: **Growth impact of strains carrying genomic- and plasmid-actuated DNAi devices.**
(A) Doubling time (in min) as a function of inducer (0 – 10 mM Ara) for host strains carrying a Y+X CRISPR plasmid (pCR-XY, Cm^R) and either the 3x DNAi device or no actuator (actuator loci deletions still present). Samples are prepared as in Figure 3a (Methods), except that washed cultures are diluted to OD_{600} = 0.01 into non-selective (-spectinomycin) 2YT (2.4 ml) containing choloramphenicol and then split into 12x200 µl samples in 96-well format in an optically-clear plate. Each sample is induced with Ara (0 – 10mM) and then grown in a BioTek Synergy H1 Hybrid Microplate Reader for 4 hr at 37°C and 560 rpm with concomitant measurement of each sample's absorbance (600nm) every 3 min. The exponential-phase growth rate, µ (in min^{-1}), is then determined by least-squares fit of measured OD_{600} values corresponding to the interval between 60 and 90 min post-induction using the equation $OD_{600} = A_0 e^{-\mu t}$, where *t* is time in minutes, and A_0 is the OD_{600} at *t* = 0. The doubling time, τ (in min), is then calculated as $\tau = \ln(2)/\mu$. **(B)** The cell density as measured by OD_{600} is shown as a function of inducer (0 – 10 mM Ara) for cells containing either the 1x DNAi device, the 3x DNAi device, or the plasmid-borne pACT-04 actuator, along with the X CRISPR targeting array (pCR-X, Cm^R), and a target plasmid (pTAR(S), RFP^+, Str^R). Samples are prepared as in (A) except that cultures (500 µl) are induced and grown in 96-well format at 37°C and 900 rpm for 8 hr, and that endpoint measurements of OD_{600} are made in optically-clear 96-well plates (100 µl sample volume) using a BioTek Synergy H1 Hybrid Microplate Reader. Arrows indicate 2 mM arabinose, the inducer concentration at which all plasmid knockout and cell killing experiments are performed. All data represent the average of three independent experiments performed on different days and the error bars are the standard deviation.

Supplementary Figure 11

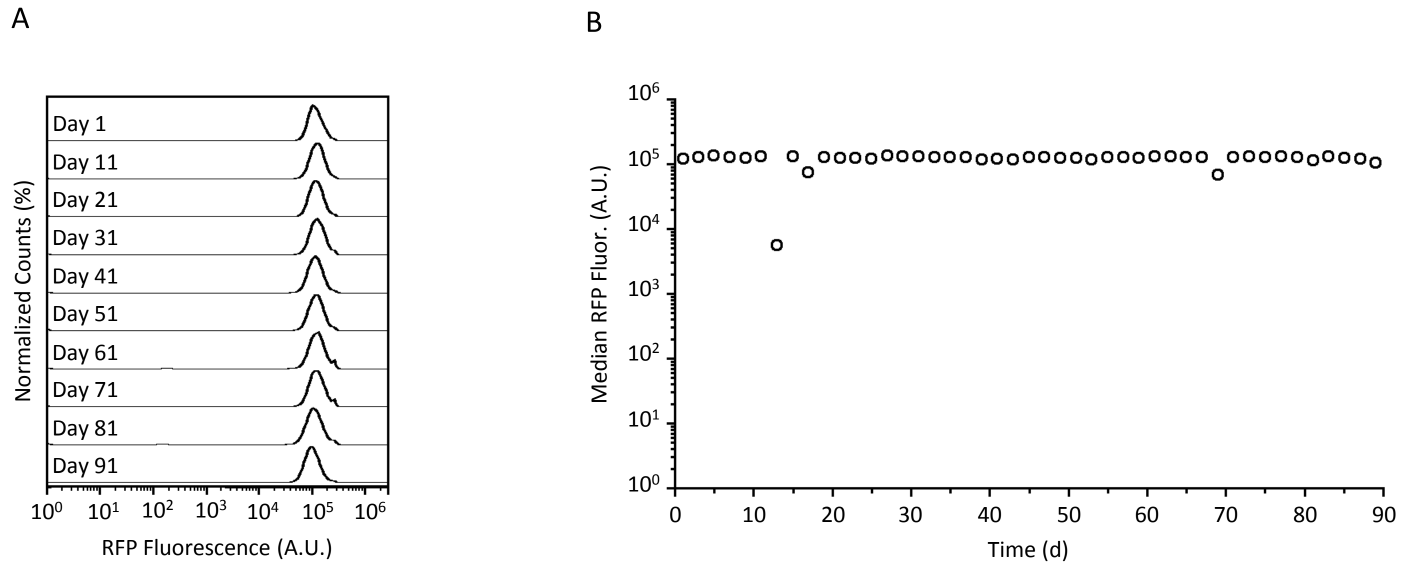

<u>Supplementary Figure 11</u>: Stability of target plasmid protein expression during cell passaging experiments.
(A) An overlay of RFP fluorescence histrograms corresponding to single cytometry measurements of a host cell sample containing the 3x DNAi device, a Y+Z CRISPR (pCR-YZ, Cm^R), and an RFP^+ target plasmid (pTAR(S), Str^R) taken every 10 days as the sample is repeatedly passaged for 90 days in the DNAi OFF state (+0.5% glucose) (Methods). Each histogram comprises >10,000 cells. **(B)** Median RFP expression level for all samples taken every two days during the experiment described in (A). Outlier data points were due to technical error.

Supplementary Figure 12

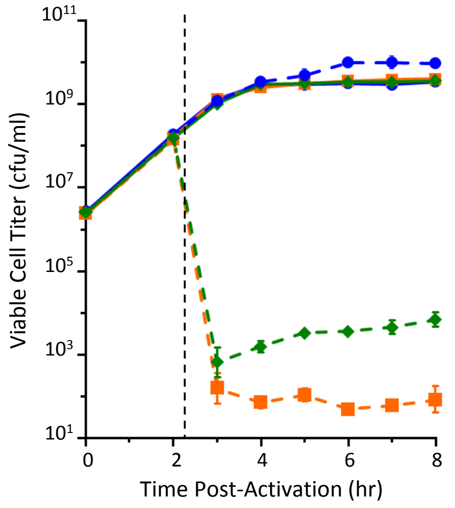

<u>**Supplementary Figure 12**</u>**: Cell killing dynamics upon targeting of Cas activity to the genome.**
The data from Figure 4b, in which host strains containing a 3x DNAi device and either one of the chromosome-targeted (G_1, green diamonds; G_2, orange squares) or the off-target (N, blue circles) CRISPRs were either induced with 2 mM arabinose (DNAi ON, dashed lines) or repressed with 0.5% glucose (DNAi OFF, solid lines) for 8 hr (Methods), is presented in terms of viable cells titer. The vertical dashed line indicates t = 2.25 hr, the time at which the P_{BAD} promoter activates to initiate the synthesis of DNAi actuator components. All data represent the average of three independent experiments performed on different days, and error bars indicate the standard deviation.

Supplementary Table 1: CRISPR element sequences

Element	Sequence	PAM	Target
Spacer G_1	CACCCGACGCCATTCTATACCTGATAATTCTT	AAG	N.C. [a] (genome)
Spacer G_2	TTGTCAACGCTCCCAGGCCGTCCACTCCCTGA	AAG	attL[b]
Spacer N_1	TGGTCTTCAGCCTTCTAGAGATATGAAGACTT	n/a	none
Spacer N_{2A}[c]	CTTTCGCAGACGCGCGGCGATACGCTCACGCA	n/a	none
Spacer N_{2B}[c]	GACTCACCCCGAAAGAGATTGCCAGCCAGCTT	n/a	none
Spacer X	ACAGTAAGAGAATTATGCAGTGCTGCCATAAC	ATG	N.C. [a] (plasmid)
Spacer Y	ACATTCTTGCAGGTATCTTCGAGCCAGCCACG	ATG	Str^R
Spacer Z	CCACACAGTGATATTGATTTGCTGGTTACGGT	AAG	Str^R
Repeat	GAGTTCCCCGCGCCAGCGGGGATAAACCG	–	–

a. N.C. = Non-coding sequence

b. Att sites correspond to Int5 recombinase used for actuator chromosomal integration

c. Spacers designated N_{2A} and N_{2B} in this work correspond to spacers with designations N_1 and N_4 from pWUR477 from Brouns et al. Science, 2008.

Supplementary Table 2: Host chromosomal modifications

Locus	Native Sequence Deleted[a]	Actuator[b] (1x strain)	Actuator[b] (3x strain)
Δ(CRISPR-cas)	2,879,926 – 2,889,743	pACT-01	pACT-02
Δ(araC-araBAD)	65,854 – 71,265	none	pACT-02
ΔLacI	366,428 – 367,586	none	pACT-01

a. Numbering based on NCBI entry for E. coli *str. K-12 substr. MG1655 (NC_000913.3)*
b. All actuators are oriented with respect to the chromosomal (–) -strand

Supplementary Table 3: Genomic modification primers

Primer[a]	Sequence[b]
Δ(CRISPR-cas3)-fwd	5'– TATCAGGTATAGAATGGCGTCGGGTGCTTGAGGCTGTCTGgtgtaggctggagctgcttc
Δ(CRISPR-cas3)-rev	5'– AATGCTACCTCTGGTGAAGGAGTTGGCGAAGGCGTCTTGAattccggggatccgtcgacc
Δ(araC-araBAD)-fwd	5'– GCTACTCCGTCAAGCCGTCAATTGTCTGATTCGTTACCAAgtgtaggctggagctgcttc
Δ(araC-araBAD)-rev	5'– AGCCTGGTTTCGTTTGATTGGCTGTGGTTTTATACAGTCAattccggggatccgtcgacc
ΔLacI-fwd	5'– TCTGGTGGCCGGAAGGCGAAGCGGCATGCATTTACGTTGAgtgtaggctggagctgcttc
ΔLacI-rev	5'– TGCCTAATGAGTGAGCTAACTCACATTAATTGCGTTGCGCattccggggatccgtcgacc

a. Forward and reverse designations are given with respect to actuator orientation following integration

b. Underlined sequence corresponds to homology (40 bp) with host chromosome. Lower case sequence corresponds to priming regions present within pKD13-AttB plasmid for amplifying the knockout cassette

Supplementary Table 4: Int5-specific recombination sites

Site	Sequence[a]
attB	GAGCGCCGGATCAGGGAGTGGACGGCCTG<u>GG</u>AGCGCTACACGCTGTGGCTGCGGTCGGTGC
attP	CCCTAATACGCAAGTCGATAACTCTCCTG<u>GG</u>AGCGTTGACAACTTGCGCACCCTGATCTG
attL	GAGCGCCGGATCAGGGAGTGGACGGCCTG<u>GG</u>AGCGTTGACAACTTGCGCACCCTGATCTG
attR	CCCTAATACGCAAGTCGATAACTCTCCTG<u>GG</u>AGCGCTACACGCTGTGGCTGCGGTCGGTGC

a. All sequences given in the forward orientation. Underlined bases corresponds to recombination point.

Supplementary Table 5: qPCR primer sets

Set	Dist. to G_1[a]	Forward primer	Reverse primer	Amplicon length	Mol. Wt. (ng/mol)
A	0	CGAGCAGCATTCCTGATTTTA	CTCGAGGAAGCAGCTCCA	110 bp	6.80×10^{13}
B	1	GGCGTCGCTTGATGAACTGATA	CCGCACGCACCGTAAAGT	83 bp	5.11×10^{13}
C	10	GCGCATTTTGATTGGCATT	CCCAGCAAAATACCGCGAT	99 bp	6.11×10^{13}
D	100	GCAATTTTTCCCGGCAAAATTACAA	CATGTTAAGAACCTCCCTATCCGTATATAA	100 bp	6.16×10^{13}
E	1000	GGTTGATCCAAGCTTCCTGACA	AAGAATGGCTGGGATCGTGGGTT	94 bp	5.80×10^{13}
Str^R	–	AAAACAAAGTTAAACATCATGAGGGAA	GATGACGCCAACTACCTCTGATA	78 bp	4.81×10^{13}

a. Center-to-center distances (in kbp) between the corresponding qPCR amplicon and the protospacer G_1 sequence (NCBI position 2,887,466) as measured along the host chromosome in the (+)-strand direction.

Supplementary Table 6: Constitutive promoter sequences

Promoter	Sequence[a]
P_{J23100}	*TTGACG*GCTAGCTCAGTCCTAGGTACAGTGCTAGc
$P_{J23100*}$ [b]	*TTGACG*gctagctcagtcctaggTAC**TA**TGCTAGc
P_{J23101}	*TTTACA*gctagctcagtcctaggATAATAGCTAGc
P_{J23117}	*TTGACA*gctagctcagtcctaggGATTGTGCTAGc

a. Italicized and underlined sequences correspond to promoter -35 and -10 elements, respectively. Lowercase base corresponds to the promoter -1 position.

b. Mutations in -10 element relative to P_{J23100} are given in bold

Supplementary Table 7: RBS sequences

Plasmid	ORF	RBS Sequence[a]
pACT-01/02	*cas3*	**A**TACCCGTTTTTTTGGGCTAGCAATAAGGAGATATACCATG
pACT-01/02	*casA*	**G**CACTCGAGTGTAGAAATAATTTTGTTTAACTTTAATAAGGAGATATACCATG
pACT-01[b]	*araC*	**G**TTTTTGTCATGGCTTTGGTCCCGCTTTGTTACAGAATGCTTT...TTCTTCTCTGAATGGCGGGAGTATGAAAAGTATG
pACT-02[d]	*araC*	**T**ACTAGAGAAAGAGGAGAAATACTAGATG
pTAR	*RFP*	**T**TTCCCCCGGAAACCAATAAAAGAAGGCCATCGTCATG
pINT5$_{ts}$	*Int5*	**A**TACCCGTTTTTTTGGGCTAGCAGGGACAGGATATGTGAGTAAACGTCGTTATCTTACCGGTAAAGAAGTTCAGGCCATG
pINT5$_{ts}$[b]	*araC*	**G**TTTTTGTCATGGCTTTGGTCCCGCTTTGTTACAGAATGCTTT...TTCTTCTCTGAATGGCGGGAGTATGAAAAGTATG
pGFP-01/02[c,d]	*GFP*	**C**CTGTcACCGGATGTGCTTTCCGGTCTGATGAGTCCGTGAGGACGAAACAGCCTCTACAAATAATTTTGTTTAA*AAAGAGGAGAAATACTAG*ATG
pJ23101-GFP[e]	*GFP*	**T**TACTAG*AGTCACACAGGAAAGTACTAG*ATG

a. RBS defined as the entire 5' UTR spanning the transcriptional +1 site (bold) through the start codon (underlined)
b. Native araC RBS sequence corresponding to E. coli *str. K-12 substr. MG1655 (NC_000913.3 positions 70225-70389)*
c. RBS includes RiboJ self-cleaving ribozyme. RNA auto-cleavage occurs 3' to the lowercase cytosine.
d. BBa_0034 RBS sequence is in italics
e. BBa_0032 RBS sequence is in italics

Supplementary Table 8: Classification of DNAi survivor LOF mutations via genetic complementation

Original pCisTAR(S)	DNAi activity observed +pACT-03/pCR? (Yes/No)					LOF assignment[a]
	+pACT-03	+pCR-N	+pCR-X	+pCR-Y	+pCR-Z	
pCisTAR(S)-X	Yes	Yes	Yes	Yes	Yes	No LOF
	Yes	No	No	No	No	Actuator
	No	No	Yes	Yes	Yes	CRISPR
	No	No	No	Yes	Yes	Target
	No	No	No	No	No	Multiple mutations
pCisTAR(S)-Y	Yes	Yes	Yes	Yes	Yes	No LOF
	Yes	No	No	No	No	Actuator
	No	No	Yes	Yes	Yes	CRISPR
	No	No	Yes	No	Yes	Target
	No	No	No	No	No	Multiple mutations
pCisTAR(S)-Z	Yes	Yes	Yes	Yes	Yes	No LOF
	Yes	No	No	No	No	Actuator
	No	No	Yes	Yes	Yes	CRISPR
	No	No	Yes	Yes	No	Target
	No	No	No	No	No	Multiple mutations
pCisTAR(S)-Y+Z	Yes	Yes	Yes	Yes	Yes	No LOF
	Yes	No	No	No	No	Actuator
	No	No	Yes	Yes	Yes	CRISPR
	No	No	Yes	No	No	Targets (both)
	No	No	No	No	No	Multiple mutations

a. While the chart does not cover all 32 Yes/No combinations for each pCisTAR(S) variant, none of the 27 other ambiguous cases were observed
